# Structures of DNA-bound human ligase IV catalytic core reveal insights into substrate binding and catalysis

**DOI:** 10.1038/s41467-018-05024-8

**Published:** 2018-07-06

**Authors:** Andrea M. Kaminski, Percy P. Tumbale, Matthew J. Schellenberg, R. Scott Williams, Jason G. Williams, Thomas A. Kunkel, Lars C. Pedersen, Katarzyna Bebenek

**Affiliations:** 1 Genome Integrity and Structural Biology Laboratory, National Institute of Environmental Health Sciences, National Institutes of Health, Research Triangle Park, 27709 NC USA; 2 Epigenetics & Stem Cell Biology Laboratory, National Institute of Environmental Health Sciences, National Institutes of Health, Research Triangle Park, 27709 NC USA

## Abstract

DNA ligase IV (LigIV) performs the final DNA nick-sealing step of classical nonhomologous end-joining, which is critical for immunoglobulin gene maturation and efficient repair of genotoxic DNA double-strand breaks. Hypomorphic LigIV mutations cause extreme radiation sensitivity and immunodeficiency in humans. To better understand the unique features of LigIV function, here we report the crystal structure of the catalytic core of human LigIV in complex with a nicked nucleic acid substrate in two distinct states—an open lysyl-AMP intermediate, and a closed DNA–adenylate form. Results from structural and mutagenesis experiments unveil a dynamic LigIV DNA encirclement mechanism characterized by extensive interdomain interactions and active site phosphoanhydride coordination, all of which are required for efficient DNA nick sealing. These studies provide a scaffold for defining impacts of LigIV catalytic core mutations and deficiencies in human LIG4 syndrome.

## Abstract (web summary)

DNA Ligase IV (LigIV) catalyzes nick sealing of DNA double-strand break substrates during non-homologous end-joining. Here the authors present the crystal structures of two human LigIV DNA-bound catalytic states, which provide insights into its catalytic mechanism and the molecular basis of LIG4 syndrome causing disease mutations.

## Introduction

DNA ligases are an essential component of biological processes involved in genome replication, recombination, and repair that rejoin DNA backbone breaks by fusing termini containing a 3′-hydroxyl and a 5′-phosphate to form a new phosphodiester bond (reviewed in ref. ^1^). All ligases employ a multi-step catalytic cycle, wherein an active site lysine attacks the α-phosphate moiety of either ATP or NAD^+^ to form a covalent enzyme-AMP intermediate (Step 1), followed by release of either pyrophosphate (for ATP-dependent ligases) or nicotinamide mononucleotide (for NAD^+^-dependent ligases) (Supplementary Fig. 1). Upon binding of a nicked oligonucleotide, the AMP moiety is transferred from the active site lysine to the 5′-phosphate end of the substrate (Step 2). In the final ligation step, the 3′-OH on the upstream side of the backbone break attacks the phosphorous atom of the 5′-phosphate, releasing AMP, and forming the new phosphodiester bond (Step 3).

Mammalian cells contain three DNA ligases (Lig)—LigI, LigIII, and LigIV. LigI fuses Okazaki fragments during DNA replication, and also plays a role in long-patch base excision repair (BER)^2^. LigIII participates in short-patch BER and single-strand break repair in the nucleus, and it is critical for maintenance of mitochondrial DNA^3–5^. LigI and LigIII have also been found to participate in the alternative microhomology-mediated double-strand break (DSB) repair pathway^6^. Meanwhile, LigIV catalyzes the ligation step during repair of DSBs through classical nonhomologous end-joining (NHEJ)^7^ and in V(D)J recombination that is critical for immunoglobulin gene maturation^8^. Additionally, cellular DNA ligases can be “hijacked” during retroviral infection (e.g., HIV and HSV-1), and may contribute to replication, processing, and integration of the viral genome^9–11^. Though some functional overlap exists between the different ligases, all three enzymes are essential for survival, as evidenced by the fact that transgenic knockout mouse models of each ligase are embryonic lethal^12–14^. While deficiency of LigIII has not yet been associated with human disease, its targeted inactivation in animals causes mitochondrial dysfunction and reduced mtDNA genome integrity^4,15^. LigI deficiency contributes to growth retardation and immunodeficiency in human patients^16^. A variety of deletions and hypomorphic mutations in LigIV have been associated with multiple systemic conditions referred to as LIG4 Syndrome, including microcephaly, developmental delay, severe combined immunodeficiency, neoplasms, and sensitivity to ionizing radiation^17^.

**Fig. 1:**
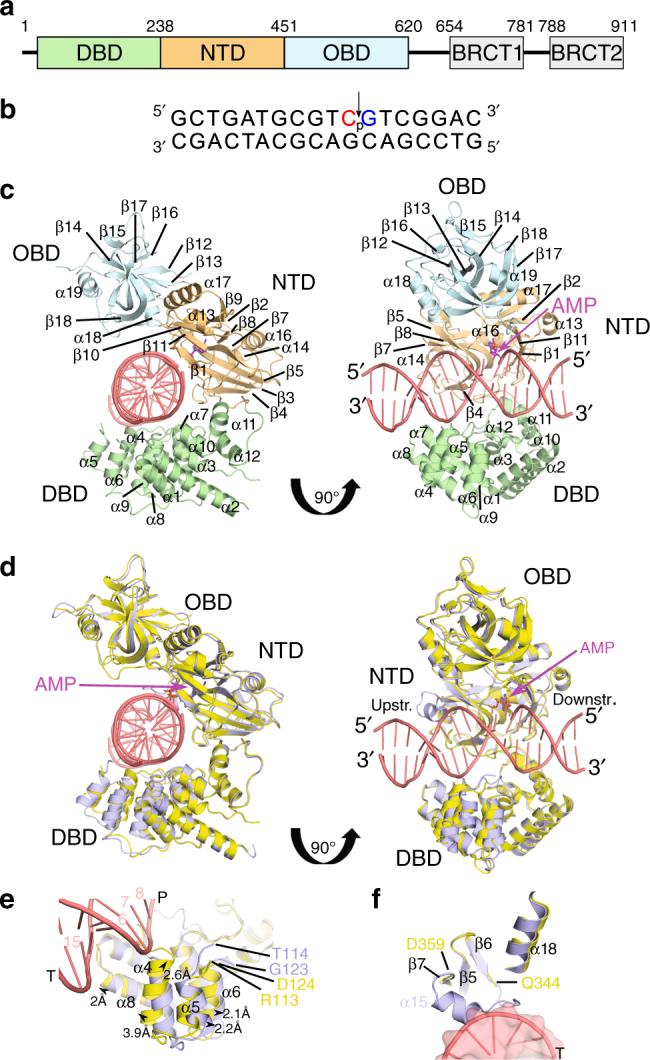
Structural characterization of the lysyl-adenylate form of the human DNA LigIV catalytic domain. **a** Schematic of human LigIV subdomain architecture. **b** Nicked DNA substrate co-crystallized with LigIV, with the backbone discontinuity indicated (arrow). The primers upstream and downstream of the nick were dideoxy-terminated (red) or phosphorylated (blue), respectively. **c** Ribbon diagram of the lysyl-adenylate (magenta) form of LigIV (DBD in green; NTD in orange; OBD in blue) bound to the nicked DNA substrate (pink), as viewed down the axis of the DNA helix (left). On the right, the complex has been rotated 90° around the *y*-axis, to view the DNA. In this orientation, the nicked DNA substrate is observed in the same orientation as in **b**, with the upstream duplex on the left, and the downstream duplex on the right. The relative positions of the protein subdomains, along with the secondary structural elements, are labeled as in Supplementary Fig. 3. **d** Comparison of the DNA-bound, lysyl-adenylate form of LigIV (protein in yellow; DNA in pink; AMP in magenta), with that of the apoprotein (protein in light blue; ATP in green; PDB ID code 3W5O^20^). Orientations are the same as in **c**. **e** Structural differences observed in the DBD of the apoprotein (light blue, PDB ID code 3W5O^20^) vs. the DNA-bound lysyl-adenylate complex (protein in yellow, DNA in pink). Direction and measured distances for movement of paired atoms are shown as black arrows. **f** Binding of a nicked DNA substrate (pink) to the catalytic domain of LigIV (yellow) results in unraveling of α-helix 15 observed in the apoprotein (light blue)

The mammalian DNA ligases share a conserved catalytic core domain architecture comprised of three subdomains—the DNA-binding (DBD), nucleotidyltransferase (NTD), and oligonucleotide/oligosaccharide-fold (OBD) subdomains^1^ (Fig. 1a). Crystal structures of the catalytic cores of human LigI and LigIII reveal that the three subdomains work in tandem, encircling the DNA substrate and correctly positioning the backbone nick at the active site^18,19^. To date, only crystal structures of the LigIV apoprotein have been reported, which were observed in an extended DNA-free open conformation^20^. To better understand the unique features of LigIV, we obtained two crystal structures of protein-DNA complexes in the ligation pathway—the lysyl-AMP intermediate in an open conformation, and the DNA–adenylate in a closed conformation. Guided by these structures, we engineered several active site and surface mutants to probe their roles in the multi-step catalytic cycle. Results from these studies provide insights into how hypomorphic mutations fundamentally alter enzymatic structure–function relationships, and establish a molecular basis for LIG4 Syndrome.

## Results

### Characterization of DNA-bound LigIV lysyl–adenylate complex

In order to determine the structural mechanisms underlying LigIV DNA substrate binding, we co-crystallized the LigIV catalytic domain LigIV (Met1-Asp620) in the presence of a nicked duplex DNA substrate containing a 5′-phosphorylated downstream primer and a catalytically incompetent 3′-dideoxycytidine-terminated upstream primer (Fig. 1b and Supplementary Table 1). This LigIV construct was chosen for co-crystallization because C-terminal truncations beyond Lys609 were shown in a previous study to severely decrease ligation activity, compared to constructs containing Leu610-Asp620, or to the full-length protein in complex with a C-terminal truncation of XRCC4^20^. Though the longer LigIV catalytic domain construct (Met1-Asp620) exhibited higher ligation activity overall, we found that this enzyme was poorly adenylated (Supplementary Fig. 2a), as determined by mass spectrometry. The population of adenylated LigIV molecules could be increased by pre-incubation of the enzyme with ATP and MgCl_2_ before binding to DNA (Supplementary Fig. 2b–d), though not to homogeneity. In a crystallization condition containing 10 mM MgCl_2_, the enzyme binds the DNA substrate in an open conformation (Fig. 1c) similar to that observed in the previously reported structure without DNA bound (LigIV Met1-Lys609 with bound ATP, PDB ID code 3W5O^20^, henceforth referred to as the apoprotein). The two structures superimpose relatively well (Fig. 1d), with an RMSD of 1.04 Å over 551 Cα atoms. In this open conformation, substrate binding is mediated primarily by the DBD and NTD subdomains, and the OBD does not directly interact with the DNA (Fig. 1c).

A detailed comparison of these two structures shows that key DNA-binding regions on the protein surface differ between the free and DNA-bound states. First, DBD α-helices 4–6 and α8 engaging the upstream DNA duplex (5′ to the nick) shift by 2.1–3.9 Å to interact with the substrate (Fig. 1e), while the other DBD secondary structural elements remain comparatively static. Helix α4, and the loop immediately preceding it, cradle the DNA phosphate backbone of the upstream strand (P6-8). DBD α8 shifts 2 Å along its axis, with the N-terminal end of the helix and the helix dipole oriented appropriately to bind the complementary upstream strand (T15). The α5-α6 connector loop (Insert 1^20^) is largely disordered in both structures.

The structure of the DNA-bound NTD is nearly indistinguishable from that of the apoprotein, with one notable exception. In the absence of the DNA substrate, β-strands 6 and 7 are linked by an α-helix (α15) spanning Ile351-Asp357 (Supplementary Fig. 3 and Fig. 1f). However, this helix would clash with the upstream duplex when DNA is bound. Thus, this region is displaced and becomes disordered (Lys345-Ser358) upon substrate binding. The loop between β-strands 12–13 (Motif Va^21^) is also poorly defined in the electron density and adopts different conformations in DNA-bound and free structures. The loop between β-strands 13 and 14 (Insert 2^20^) is ordered in the apoprotein structure, but not in the DNA-bound structure.

### Nick “sensing” by LigIV

**Fig. 2:**
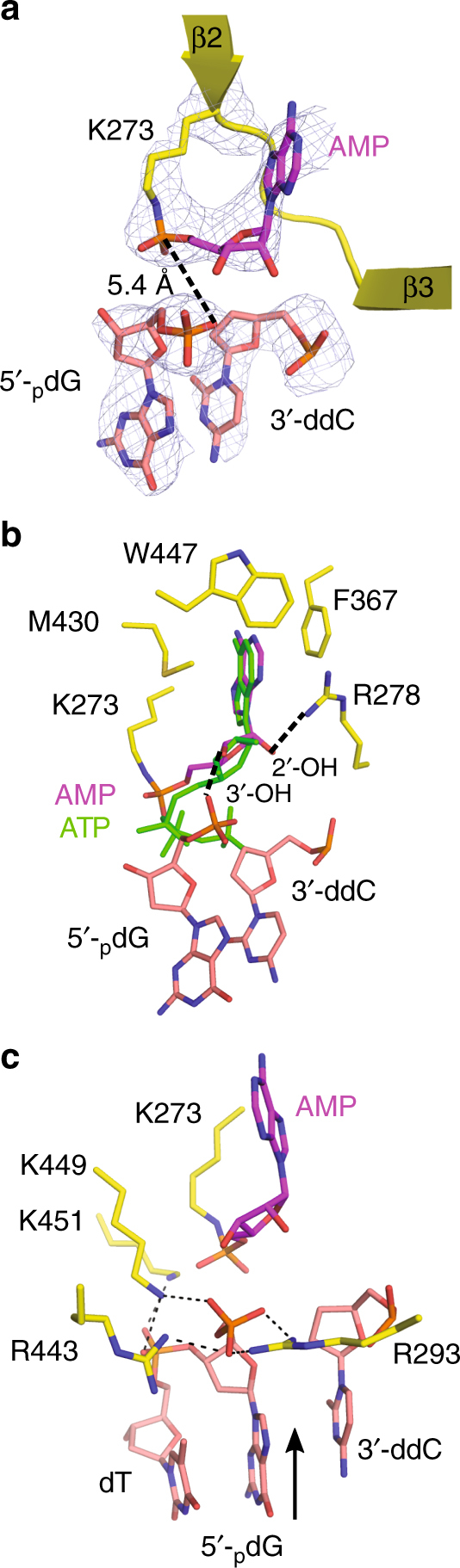
Detailed analysis of the lysyl-adenylate complex. **a** Disposition of the LigIV lysyl-adenylate (magenta) covalently linked to Lys273, relative to the nick in the DNA backbone (pink). The interatomic distance between the oxygens on the 5′-phosphate on the downstream primer and the phosphorous atom of the AMP is indicated by the black dashed line (~5.4 Å). A simulated annealing omit *F*_o_ − *F*_c_ difference electron density map (gray) is shown, contoured at 2.5*σ*. **b** Interactions with the lysyl-adenylate (magenta) and the protein residues (yellow) lining the nucleotide-binding site. Putative hydrogen-bonding interactions with the AMP hydroxyls are indicated by black dashed lines, and are listed in detail in Supplementary Table 2. The LigIV apoprotein structure (PDB ID code 3W5O^20^) was superimposed onto the DNA-bound structure, and the position of its ATP molecule is shown in green. **c** Nick sensing by LigIV involves multiple putative interactions (black dashed lines) between protein sidechains (yellow), the adenylate (magenta), and the 5′-phosphate on the DNA (pink). The nick junction is indicated by a black arrow

At 3.25 Å resolution, the electron density for the bound nicked DNA substrate and for the adenylate group covalently linked to the sidechain of Lys273 is clearly defined (Fig. 2a). The adenylate is stabilized through hydrophobic stacking interactions with Phe367 and Met430 within the conserved AMP-binding pocket in the NTD (Fig. 2b and Supplementary Fig. 4a). The AMP 3′-hydroxyl lies in close proximity to the 5′-phosphate oxygens on the downstream primer, while the 2′-OH lies within hydrogen-bonding distance of the sidechain of Arg278 (Supplementary Fig. 4a and Supplementary Table 2). The position of the lysyl-adenylate is similar to that of the ATP molecule modeled in the nucleotide-binding site of the apoprotein (PDB ID code 3W5O^20^) with regard to the base, but deviates in the arrangement of its 5′-phosphate (Fig. 2b). In the DNA-bound lysyl-adenylate structure, the nearest 5′-phosphate oxygen on the downstream primer lies ~5.4 Å away from the phosphorus atom of the AMP (Fig. 2a). Thus, structural repositioning of the 5′-phosphate and/or the lysyl-AMP would be required to facilitate nucleophilic attack and the adenyl-transfer to the DNA.

LigIV senses the nick through binding of the 5′-phosphate, which is positioned appropriately at the active site. The 5′-phosphate oxygens lie within hydrogen-bonding distance of both Arg293 and Arg443 (Fig. 2c, Supplementary Fig. 4a, and Supplementary Table 2). Additionally, Lys449 is positioned to potentially interact with both the 5′-phosphate and the next backbone phosphate downstream from the nick. Lys451 also lies within hydrogen-bonding distance of that downstream phosphate. The high density of positive charge in this region likely serves to offset the negative charges present on the AMP and the 5′-phosphate.

### DNA–adenylate complex of LigIV adopts a closed conformation

**Fig. 3:**
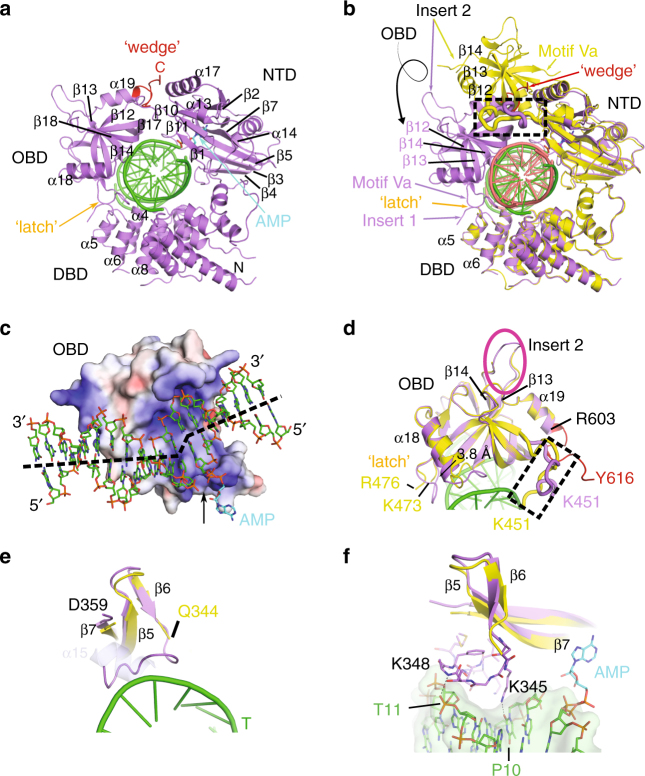
The DNA–adenylate complex adopts a catalytically competent closed conformation. **a** Ribbon diagram of the LigIV catalytic domain (purple) bound to a nicked DNA substrate (green), where the adenylate group (cyan) has been covalently transferred from Lys273 to the 5′-phosphate on the downstream strand. The complex is viewed down the axis of the DNA helix. **b** Superposition of the open lysyl-adenylate (protein in yellow, DNA in pink) and closed DNA–adenylate (protein in purple, DNA in green) complexes of LigIV. The hinge region is marked with a black dashed box. During the open to closed transition, the OBD domain undergoes a complex “swivel-and-close” motion to encircle the DNA, the direction of which is indicated by the spiral arrow. Secondary structural elements in ribbon diagrams are labeled as in Supplementary Fig. 3. The ordered region of the C-terminal tail in the closed DNA–adenylate complex is shown in red and the ‘latch’ is indicated in orange. **c** Rendering of the electrostatic surface of the OBD subdomain in the closed conformation, with the bound nicked DNA–adenylate substrate (DNA in green, adenylate in cyan). The electrostatic surface potential was calculated using the Adaptive Poisson–Boltzmann Solver tool in PyMOL^50^, which ranges from −6 kT e^−1^ (electronegative, red) to 6 kT e^−1^ (electropositive, blue). Regions of neutral charge are shown in white. The nicked DNA substrate (green) with the 5′-adenylate (cyan) is drawn in stick. The nick junction is indicated by a black arrow. The trajectories of the upstream and downstream duplex are indicated by a dashed black line. **d**, **e** Detailed aspects of conformational differences between the open (yellow) and closed (protein in purple, DNA in green) conformations in the OBD subdomain (**d**, linker between NTD and OBD delineated by a black dashed box, and Insert 2 by a magenta oval) and the loop between β6 and β7 (**e**). α15 observed in the apoprotein structure (PDB ID 3W5O^20^) is drawn transparently in gray. **f** Detailed view of the interactions of the β6-β7 connecting loop with the DNA substrate. Putative hydrogen-bonding interactions with the DNA substrate are shown as black dashed lines

In a second DNA-bound crystal form, we trapped the LigIV catalytic domain bound to adenylated DNA, in a catalytically competent closed conformation poised for nick sealing (Supplementary Table 1 and Fig. 3a). Structural superposition with the open LigIV/DNA complex shows that the structures of the DBD–NTD subdomains align well (RMSD of 0.78 Å over 395 Cα atoms), suggesting they may function as a rigid unit (Fig. 3b). The OBD subdomain undergoes a complex swivel-and-close conformational change that allows LigIV to completely encircle the DNA in a conformation similar to those observed in the DNA-bound structures of human DNA Ligases I^19^ and III^18^. The open/closed transition is initiated by a substantial conformational change in the hinge between β11 of the NTD and β12 of the OBD (Motif V^19^) (Fig. 3b, black dashed box). Although its position changes dramatically with respect to the DBD–NTD scaffold and the DNA substrate, the global structure of the OBD remains largely unchanged (RMSD of 0.27 Å over 109 Cα atoms). Unlike the open LigIV DNA-bound conformer, where the OBD does not directly engage the substrate, the transition to the closed conformation repositions the OBD’s electropositive surface, forming extensive interactions with the DNA substrate (Fig. 3c). The curve of the central OB-fold β-sheet nestles into the minor groove of the duplex DNA downstream of the nick, shifting the N-terminal end of α18 by 3.8 Å to accommodate DNA substrate binding and altering the conformation of the loop connecting it to β14 (Fig. 3d).

Upon closure of the ring in the DNA-bound state, some residues in the NTD β6-β7 connecting loop (Lys345-Met354) become ordered, coinciding with unfolding of α15 (Fig. 3e). This loop follows the contour of the upstream strand, and only a single long-range putative hydrogen bond is observed between the backbone amide of Lys348 and the phosphate oxygen of T11 (Fig. 3f). The sidechain of Lys348 is disordered in this structure, though that of Lys345 extends into the minor groove and makes a hydrogen bond with the O2 atom on the base of residue P10 (Fig. 3f, Supplementary Fig. 4b, and Supplementary Table 2). This interaction is not likely to confer sequence specificity, since a hydrogen bond is possible at this position with any purine or pyrimidine base.

### Structural features unique to LigIV

**Fig. 4:**
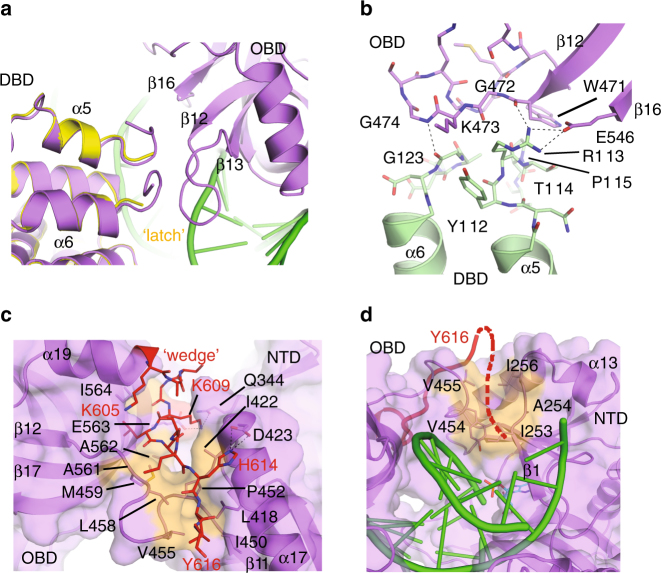
Striking structural features of the LigIV catalytic domain. **a** Comparison of the “latch” (orange) in the open (yellow) and closed DNA–adenylate complex (protein in purple, DNA in green). **b** Details of “latch” interactions between the DBD (green) and OBD (purple) subdomains, with protein sidechains drawn in stick. **c** Disposition of the “wedge” motif. The C-terminal tail (red) becomes ordered in the closed conformation, and covers a hydrophobic channel (orange) between the OBD and NTD subdomains, the potential trajectory of which continues along the protein surface toward the downstream DNA (green) duplex (**d**). Putative hydrogen-bonding interactions are indicated by black dashed lines. A detailed list of “latch” interactions is given in Supplementary Table 3

Comparison of the LigIV open and closed conformers identified three striking structural features. The β13–β14 connecting loop (Insert 2) becomes ordered in the closed conformation. Though this loop was hypothesized in a previous study to clash with a bound oligonucleotide^20^, its position in the closed conformation is far from the DNA (Fig. 3b). The β12–β13 connecting loop (Motif Va) in the OBD also becomes ordered in the closed conformation, effectively forming a “latch” with Insert 1 of the DBD, helping to bridge the two subdomains (Fig. 4a). The DBD–OBD interface is compact (292 Å^2^, as calculated by PISA^22^), and is largely mediated through a small number of backbone hydrogen bonds and van der Waals contacts (Supplementary Table 3). A salt bridge is observed between Arg113 in the DBD and Glu546 in the OBD (Fig. 4b).

The most notable difference between the DNA-bound conformers is observed at the extreme C-terminal end of the OBD, where residues Gly604-Tyr616 that were disordered in the open conformer become ordered in the closed conformation, which stabilizes the NTD–OBD interdomain quaternary structure. Gly604 and Lys605 extend α19, after which this tail then continues as a coil to Tyr616, following a narrow channel between the NTD and the OBD (Fig. 4c). This channel is lined with predominantly hydrophobic residues, including Leu418 and Ile422 from α17; Ile450 from the end of β11; Pro452, Val455, Leu458, and Met459 from the β11-β12 linker; and Ala561, Ala562, and Ile564 from β17 in the OBD (Fig. 4c). The C-terminal tail covers this hydrophobic region, sequestering it from solvent, and reinforcing the NTD–OBD interface. Additionally, there are putative hydrogen-bonding interactions between Lys609 and Gln344 (3.0 Å) and Glu563 (2.7 Å), and between His614 and Asp423 (2.9–3.0 Å). In this conformation, the C-terminal tail might “wedge” the OBD into the closed conformation, preventing premature opening of the OBD subdomain. This hypothesis is consistent with the observation that LigIV constructs truncated at Lys609 exhibit decreased end-joining and concatamerization of a sticky-end substrate^20,23^. It should be noted that, although the C-terminus is disordered after Tyr616, the contour of the hydrophobic cleft continues between the β1-α13 connecting loop and the NTD–OBD linker (Fig. 4d). If the C-terminal tail followed the trajectory of this channel, it could contact the major groove of the DNA duplex downstream of the nick. This is consistent with the presence of positively charged residues (Lys626-Lys636) in the linker between the catalytic domain and the first BRCT domain, which could contribute to DNA binding.

### Structural insights into the catalytic mechanism

**Fig. 5:**
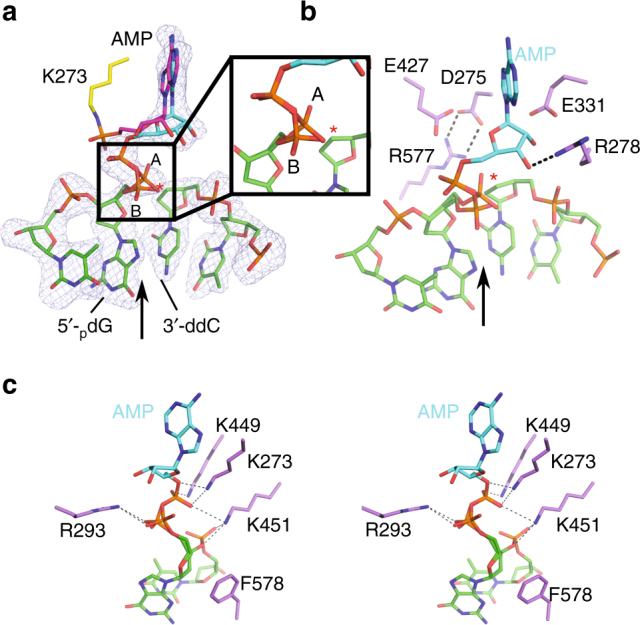
Analysis of interactions involving the DNA–adenylate prior to nick sealing. **a** The lysyl-adenylate from the open complex (K273 in yellow, AMP in magenta) was superimposed with the DNA–adenylate (AMP in cyan, DNA in green). Alternate conformations of the primer terminal 5′-phosphate are shown and indicated as “A” or “B” (magnified view in inset), and the putative position of the 3′-OH is marked with a red asterisk. A simulated annealing omit *F*_o_ − *F*_c_ difference electron density map (gray) is shown for residues in the closed DNA–adenylate complex, contoured at 2.5*σ*. **b** Candidates for divalent metal binding within the LigIV active site. The nick junction is indicated by a black arrow. **c** Stereo diagram of potential interactions involved in charge neutralization and/or substrate stabilization. A detailed list of the hydrogen-bonding interactions is given in Supplementary Table 2

In the closed conformation, there is clear density indicating that the adenylate was successfully transferred from Lys273 to the 5′-phosphate of the nicked DNA (Fig. 5a). The AMP phosphate is well-ordered, but the 5′-phosphate on the downstream primer exhibits more mobility, and has been modeled in two alternate conformations. The higher occupancy conformation (refined occupancy ~0.6) is likely to be the catalytically relevant conformation for the nick-sealing substrate conformation. We posit that the other conformer is intermediate between the product state of step 2 (adenylate transfer) and the substrate conformation for step 3 (nick sealing). Our observations are consistent with the model stemming from analysis of RNA ligase crystal structures, indicating that a rearrangement of the phosphoanhydride must occur to facilitate the transition between steps 2 and 3 of the ligation reaction^24^.

Eukaryotic ATP-dependent ligases utilize divalent metal cations, usually magnesium, to carry out nick sealing^25^. No divalent metal ions were observed in either the protein–adenylate or the DNA–adenylate structures, which may correlate with the lack of a 3′-OH on the primer terminus. Attempts to populate the metal binding site(s) by soaking the DNA–adenylate complex crystals with magnesium or manganese were not successful, and ultimately destabilized the crystal lattice. The absence of metal ions in these structures was surprising; therefore, the magnesium-dependence of LigIV activity was assayed on both unadenylated and pre-adenylated nicked substrates. Magnesium was absolutely required for nick sealing on both substrates (Supplementary Fig. 5a). We subsequently performed a metal specificity screen, using a variety of different divalent cations (Mg^2+^, Mn^2+^, Ca^2+^, Zn^2+^, Co^2+^, Ni^2+^, and Cu^2+^). All ions except zinc and copper were permissive for ligation (Supplementary Fig. 5b).

A number of interactions potentially involved in the catalytic mechanism are observed within the active site. Three candidate residues for binding divalent metal ions (Glu331, Glu427, and Asp275) lie proximal to the AMP and 5′-phosphate of the downstream strand (Fig. 5b and Supplementary Fig. 4b). In the absence of bound metal ion(s), Asp275 from the NTD is observed forming a salt bridge with Arg577 in the OBD, which could stabilize the closed conformation. The sidechain of Phe578 supports the sugar moiety of the 5′ side of the nick junction, potentially through π-CH interactions (Fig. 5c). After the transfer of the adenylate from Lys273 to the DNA, the lysine sidechain remains in close proximity to the 5′-phosphate of the AMP, as does Lys449 (Supplementary Table 2). The NZ atom of Lys451 lies in an optimal position to participate simultaneously in long-range interactions with a phosphate oxygen of the AMP, and those of the phosphate backbone (Fig. 5c). Arg293 maintains its proximity to the 5′-phosphate on residue D2 in both the open and closed complexes, while the interaction of Arg443 with the same phosphate is observed only the open conformation. The relatively large number of positively charged sidechains in this area likely serves to neutralize the dense concentration of negative charge from the phosphates during the ligation. It should be noted that interactions with the DNA substrate largely involve the region of the nick and the immediately adjacent downstream strand.

### Structure-guided mutagenesis of the LigIV active site

**Fig. 6:**
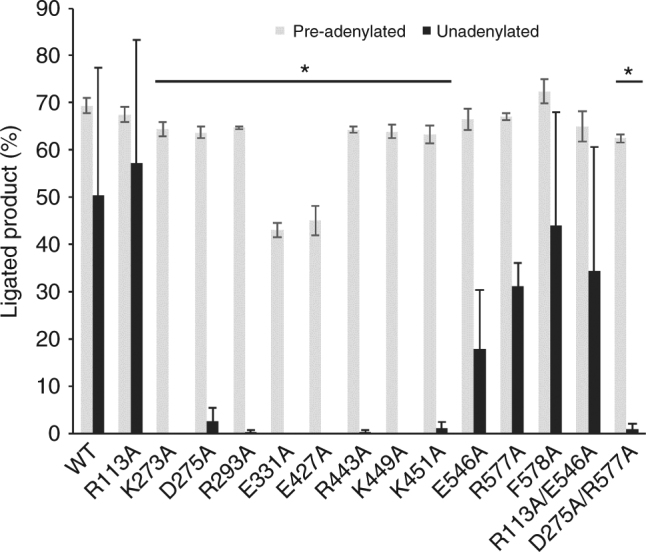
Analysis of nick ligation by the wild-type and mutant LigIV catalytic domains. Levels of ligation activity for wild type and mutant LigIV catalytic domain constructs were examined using either unadenylated (black bars) or pre-adenylated (gray bars) substrates. 1 mM ATP was included in reactions with the unadenylated substrate, to aid in enzyme adenylation, and was not included in reactions with the pre-adenylated substrate. Extent of ligation product formation for each LigIV variant is indicated, as mean ± standard deviation. (*, *p* < 0.05, WT vs. LigIV variant)

To understand the role of the various interactions immediately surrounding the nick, the crystal structures of both the open lysyl-adenylate and closed DNA–adenylate conformers were used to target key active site residues for alanine scanning mutagenesis to probe their functional roles in LigIV catalysis (Supplementary Fig. 4). Additionally, sidechains that could be involved in the closing of the “latch” (Arg113 and Glu546) were also investigated. Interactions involving multiple residues were evaluated singly and in pairs. All mutations were generated in the same catalytic domain construct used for crystallization (Met1-Asp620), and all expressed as soluble proteins. Analysis of all recombinant WT and mutant proteins using mass spectrometry indicates inefficient lysyl-adenylation of Lys273 (Supplementary Fig. 6). The mutants were tested for nick-sealing activity, using nicked DNA substrate with a sequence context identical to that used for crystallization. Consistent with previous reports^20^, and with the apparently poor extent of adenylation in vivo, most LigIV catalytic domain variants displayed low levels of ligation activity (Fig. 6, black bars, and Supplementary Fig. 7). This activity could be augmented by including ATP in the reaction mixtures, ostensibly by increasing the amount of available adenylated enzyme. Of the ‘latch’ mutants analyzed, R113A had no effect on ligation activity and both E546A and the R113A/E546A mutants displayed only slightly decreased activity (Fig. 6). F578A was indistinguishable from the wild-type protein, indicating its stabilization of the downstream 5′-primer terminus is likely dispensable for ligation. R557A also exhibited slightly lower activity. In contrast, D275A, R293A, E331A, E427A, R443A, K449A, and K451A exhibited almost undetectable levels of nick sealing. The D275A/R577A double mutant was similarly defective, which suggests that Asp275 plays a more critical role than Arg577 in catalysis. The K273A mutant, as the receptor of the covalent adenylate modification, was unsurprisingly enzymatically inactive. To differentiate the final ligation step (Step 3, Supplementary Fig. 1) from potential issues with adenylation (Step 1) or adenylyl transfer (Step 2), the ligation capability on a nicked DNA substrate containing a pre-adenylated downstream primer was then assayed for all LigIV variants (Fig. 6, gray bars). All ligation-defective mutants regained nick-sealing activity when Steps 1 and 2 of the reaction pathway had been bypassed (Fig. 6 and Supplementary Fig. 8). The levels of ligation were noticeably higher on the pre-adenylated substrate than on the unadenylated substrate under the same conditions, indicating that formation of the enzyme-AMP intermediate and/or the transfer of the adenylate to the DNA are rate limiting, which is consistent with previously reported kinetic characterizations of LigIV^26^.

### Comparison of the mammalian DNA ligases

**Fig. 7:**
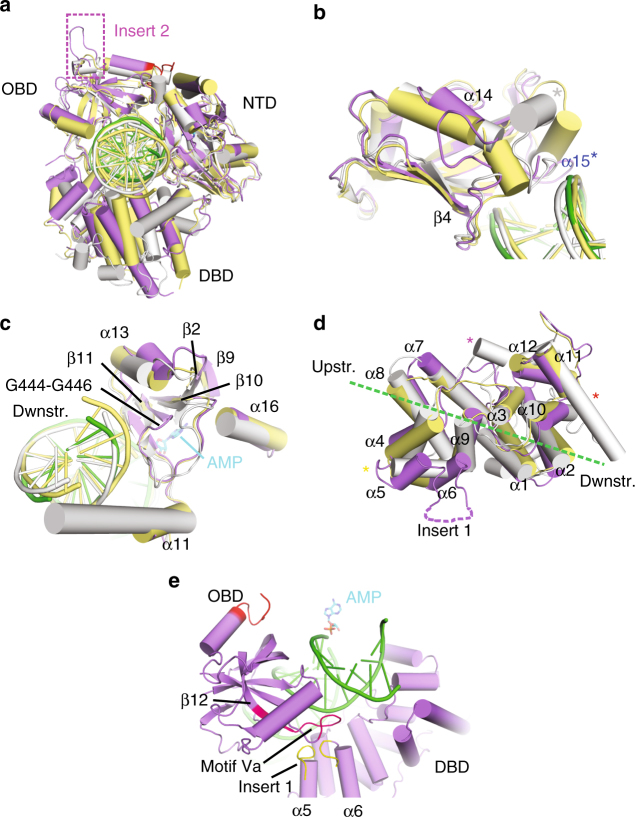
Structural comparison of the major mammalian DNA ligases. **a** Global superposition of the closed DNA-bound conformations of LigIV (purple, DNA in green), LigI (gray, PDB ID code 1X9N^19^), and LigIII (khaki, PDB ID code 3L2P^18^). Structural differences in the NTD (**b**), OBD (**c**), and DBD (**d**) subdomains of the mammalian ligases (DNA binding path in green). **e** Regions of interest in LigIV that contribute to its unique biological behaviors. Labels for secondary structural elements are representative of LigIV (numbered as in Supplementary Fig 3). Structurally disparate motifs are marked by asterisks, as follows: blue asterisk, α-helix that is conserved in LigI and LigIII structures, but is disordered (α15) in the DNA-bound structure of LigIV; gray, short α-helix present only in LigI (Asp647-Ile651); red asterisk, α11 is considerably longer in LigI than in LigIV; magenta asterisk, short helix present only in LigI (Pro341-Gly344); yellow asterisk, α5-α6 and Insert 1 regions in LigIV are dissimilar to the equivalent region in LigI and LigIII

With the successful crystallization of human LigIV in the presence of DNA, we now have substrate-bound complexes for all three DNA ligases (LigI^19^, LigIII^18^)—including two discrete catalytic states for LigIV. These structures allow for a thorough comparative analysis of ligase substrate binding and catalysis by these enzymes. All three ligases have a conserved catalytic core comprised of three subdomains (DBD, NTD, and OBD), which encircle the duplex DNA substrate and correctly position the end of the nick within the active site (Fig. 7). The structure of LigIV superimposes well globally with those of LigI and LigIII (RMSDs of 2.1 Å and 1.8 Å over 496 Cα atoms, respectively)^18,19^. The DNA binding footprint surrounding the nick is nearly identical in each case. The NTD and OBD structures of all three ligases are the most similar, with the contours of DNA binding surfaces of these domains fitting closely to the DNA (Fig. 7a). In the NTD, the α-helix cradled by the curved central β-sheet is longest and slightly kinked in LigIII, and is shortest in LigIV (α14), though the loop immediately preceding this helix is considerably longer (Fig. 7b). LigI and LigIII both contain the equivalent of α15, which is disordered in LigIV in the presence of the DNA substrate (Fig. 7b, blue asterisk). LigI also has an additional short α-helix (Asp647-Ile651) that is not present in either LigIII or LigIV (before β7 in LigIV) (Fig. 7b, gray asterisk).

There are differences in the disposition of several surface loops in the NTD and OBD subdomains, including the Insert 2 loop observed in LigIV, that is lacking in LigI or LigIII (Fig. 7a, magenta box). Conversely, LigI contains a unique insertion in the loop between α-helices 17 and 18 in LigIV, whose structure in this region is nearly identical to that of LigIII. The loop between β-strands 10 (containing Motif IV) and 11 differs between all three ligases (Fig. 7c). The position of the C-terminal end of β10 is largely conserved, and the N-terminal portion of the following loop is similar in structure between LigIV and LigIII. This loop is longer in LigI, and occupies a cleft between the long α-helix in the DBD and the phosphate backbone of the bound DNA substrate. The C-terminal end of the loop is similar for LigI and LigIII at the start of β11, but is slightly displaced in LigIV due to the small insertion of Gly444-Gly446. In spite of this small insertion, the positions of all LigIV sidechains interacting with the adenylate are structurally conserved.

The most notable differences between the three DNA ligases are observed in the DNA-binding subdomain. Though the ligases bind and encircle the DNA substrate in a similar manner, the α-helices that comprise the DBD can differ in length, placement, and orientation. The structural equivalent to α11 in LigIV is considerably longer in LigI (Fig. 7d, red asterisk), and correlates with a larger loop connecting it to the preceding helix. LigI also contains a short α-helix (Pro341-Gly344, Fig. 7d, magenta asterisk) between α3 and α4 in LigIV that is not observed in the other ligases. LigIV α5 and α6 are oriented differently from the homologous region in LigI and LigIII (Fig. 7d, yellow asterisk), which comprises a single α-helix containing Gln365-Ala383 and Asn250-Gln273 in LigI and LigIII, respectively. The α5-α6 connecting loop contains Insert 1, which has recently been found to confer the unique tolerance of LigIV for ligation of DSB ends containing damaged or mispaired bases^27^. Deletion of this loop and its replacement with a short sequence from LigIII yields similar overall ligation activity, combined with a severe decrease in damage/mismatch tolerance, similar to LigIII. The role of Insert 1 in LigIV fidelity is unclear from the structure, since it is partially disordered in the closed conformation. Its trajectory, however, places it near the DNA duplex immediately opposite the nick, forming the “latch” with Motif Va (Fig. 7e). This suggests that Insert 1 and Motif Va could work in concert to provide a sensing mechanism for distortions in canonical helical geometry, and possibly play a role in ligation fidelity by allowing for greater promiscuity on mispaired or damaged substrates.

## Discussion

**Fig. 8:**
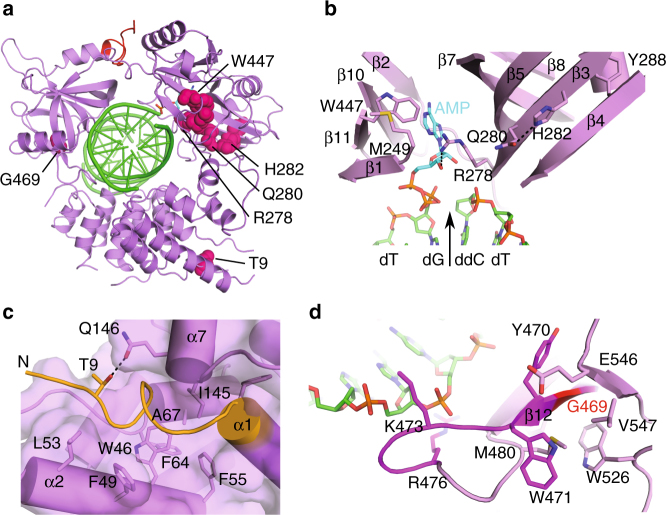
Structural insights into LIG4 syndrome mutations and polymorphisms. **a** Known LIG4 syndrome mutations (residues in space-filling representation, hot pink) mapped onto the LigIV closed DNA–adenylate structure (protein in purple, DNA in green). **b** Relative positions of mutations near the AMP (cyan) binding site of human LigIV that have been associated with LigIV deficiency. **c** Conformation of the LigIV N-terminus (orange), and its putative role in protein folding and stability. Thr9 makes a putative hydrogen bond with Gln146, which could be disrupted when mutated to isoleucine. **d** Disposition of residues in Motif Va (magenta, with Gly469 in red), in relation to the bound DNA substrate (green)

The structures presented in this study provide insight into mutations associated with LIG4 syndrome^28^ (Supplementary Fig. 3 and Fig. 8). Substitution of Arg278 with histidine confers radiosensitivity in human patients, likely due to disruption of a hydrogen bond between Arg278 and the 2′-OH of ATP, reducing its binding affinity and impairing subsequent lysyl-adenylate formation^29^ (Fig. 2b). This is consistent with the observation that some ligation activity in the R278H mutant could be regained by increasing the concentration of available ATP in the reaction mixture^29^. Disruption of this hydrogen bond could also affect the efficiency of the ligation reaction, independent of lysyl-adenylation, by decreasing the binding affinity of the mutant protein for an adenylated nicked DNA. This possibility is consistent with the lack of detectable ligation activity on this type of substrate^30^. Other mutations associated with LigIV syndrome have been identified, which exist in close proximity to Arg278. Gln280 and His282, on β3 and Tyr288 on β4 are buried within the core of the NTD, and substitutions of these residues could disrupt the structure of the central β-sheet and alter the structure of the ATP-binding pocket^31^ (Fig. 8a, b). Two mutations/polymorphisms, A3V and T9I, have been found in the N-terminus of LigIV^32^. Ala3 is disordered in our structures, but Thr9 is visible, and is the first ordered residue in the open conformation. Thr9 makes a putative hydrogen bond with Gln146 on the N-terminal end of α-helix 7 (Fig. 8c). Substitution of this residue with isoleucine would disrupt this interaction, potentially dislodging the LigIV N-terminus and exposing a hydrophobic patch between the end of α7 and the long axis of α2, which could destabilize the protein fold. A recently identified LIG4 syndrome mutation, W447C^33^, lies within van der Waals interaction distance of the AMP observed in both LigIV complexes, and is a structural component of the ATP-binding pocket (Figs. 2b and  8b). Met249 participates in a close, hydrophobic interaction with Trp447, and substitution of Met249 with a valine^34^ could alter or affect the conformation of the tryptophan sidechain, and by proxy, that of the ATP-binding pocket (Fig. 8b). Mutation of Gly469, in the OBD, to glutamate^32^ yields a poorly expressed, relatively unstable protein that has nearly undetectable levels of adenylation or ligation activity^21^. Our structures indicate that this residue lies in a tightly packed area near the C-terminal end of β-strand 12 in Motif Va, buried within the twisting β-barrel of the OBD (Fig. 8d). Substitution of this amino acid with one containing a larger, charged sidechain would be severely disruptive to the structure in this region, since it would generate steric clashes with the neighboring sidechains of Trp526, Phe483, and Met480. Consistent with this idea, alanine rather than glutamate substitutions of Gly469 were better tolerated, and yielded more adenylation and ligation activity^21^. Though they have not been associated with LIG4 syndrome, mutations of Tyr470, Arg476, and Lys473 of the Motif Va loop following β-strand 12 decreased ligation efficiency, without unduly affecting adenylation or DNA binding. These results are consistent with the observation that none of these residues directly contact the DNA backbone.

With the exception of the R278H mutation, all LIG4 syndrome mutations are thought to adversely affect protein fold and stability. These mutants are considered to be hypomorphic, and are capable of retaining a residual level of ligation activity suggested to be sufficient for DSB joining in vivo^32^. Our structure-guided mutagenesis studies give insight into the mechanism employed by LigIV, and the differential roles played by specific residues in catalysis. Arg113 and Glu546 sidechains are likely dispensable for catalysis since the R113A and R113A/E546A mutants exhibited only slightly decreased ligation activity (Fig. 6). Because none of the ligation-defective mutants appeared to generate a DNA–adenylate intermediate (except for a trace amount by K449A, Supplementary Fig 7), these mutants are likely defective in either formation of the enzyme-AMP complex (Step 1), or in adenylyl transfer (Step 2). Since the R443A, K449A, and K451A mutants have only trace amounts of ligation activity on the unadenylated substrate, but activity is rescued when the substrate is pre-adenylated, these positively charged residues likely serve to neutralize the dense negative charge on the triphosphate of the ATP cofactor, or formation of the 5′-5′ phosphoanhydride linkage during adenylation and transfer of the AMP from Lys273 to the DNA (Supplementary Figs. 1 and 4). Given the dependence of ligases on divalent metal cations^25,35,36^ (Supplementary Fig. 5a), the rescue of nick-sealing activity on a pre-adenylated DNA substrate by the E331A and E427A mutants was surprising (Fig. 6 and Supplementary Fig. 4b), since these residues were candidates for metal binding. It should be noted that although some activity was restored, the levels of activity for these mutants did not reach that of the wild-type enzyme. These residues are clearly critical for either formation of the enzyme–AMP complex, or the transfer of the AMP to the DNA substrate, but are less critical for final ligation (Fig. 6). This interpretation is supported by the observation that even trace amounts of divalent metals are sufficient to effectively promote final ligation, but are insufficient to support efficient adenylation and/or adenyl-transfer (Supplementary Fig. 5a). Another plausible explanation for this behavior is that the active sites of the ligases may have evolved to bind hydrated metals, such as those visible in the structure of the NgrRnl RNA ligase from *Naegleria gruberi*^36^, wherein all protein sidechains make second-shell hydrogen-bonding interactions with the metal through water molecules. Use of second-shell interactions rather than direct protein–metal coordination may correlate with the decreased binding affinity of LigI for Mg^2+^ during final nick sealing^35^. If this binding mode is utilized by LigIV, it is conceivable that the Glu331 and Glu427 could cooperatively coordinate the metal(s) through its hydration sphere, which could lessen the deleterious effect of mutating either residue individually. This hypothesis is consistent with the decrease, rather than total ablation of function, observed for the E331A and E427A mutations (Fig. 6).

Obtaining both the structural and biochemical data for LigIV provides a foundation for comparison of its catalytic cycle to that of other related ligases. For example, our hypothesis that Glu331 and Glu427 might aid in metal binding may be incomplete, since residues homologous to Glu331 and Glu427 behave differently in the T4 RNA ligases Rnl1 (Glu159 and Glu227) and Rnl2 (Glu99) (Supplementary Table 4). In these enzymes, substitution mutants remained largely inactive, even on pre-adenylated substrates^37–39^. Interestingly, the lysine residue accepting covalent modification of the AMP in LigIV, Lys273, exhibits a similar pattern of behavior. The homologous residue in T4 Rnl1 (Lys99) was catalytically inactive on the adenylated substrate^37^. In contrast, on pre-adenylated substrates, alanine substitution mutants of the equivalent lysine residues exhibited activity that either met (K273A in LigIV) or exceeded (K27A in *Chlorella* virus DNA ligase) that of the wild type^40^. Two key residues, Asp275 and Arg293 in LigIV, have been hypothesized in other enzymes (Asp29 in *Chlorella* ligase and Arg55 in T4 Rnl2) to play a direct role in adenylyl transfer from the enzyme to the DNA. Intriguingly, the *Chlorella* D29A and Rnl2 R55A mutants exhibited either undetectable or trace levels of activity on the adenylated substrate^39,40^, but the equivalent mutants in LigIV were proficient on this substrate. In T4 Rnl2, the equivalent residue to Asp275 is not conserved, and is observed to be a histidine (His37). When His37 is replaced with aspartate, rather than alanine, the resulting mutant exhibits activity levels similar to wild type on the adenylated substrate. Another remarkable difference appears to be in the role of Arg577 and Phe578, which behave almost identically to wild-type LigIV when substituted with alanine. In contrast, mutation of the homologous residues in LigIII and the *Chlorella* virus DNA ligase yielded little ligation activity^41,42^. It should be noted that variations in the reaction conditions may account for some of these differences between enzymes. These studies provide insights into substrate binding and catalysis by LigIV, and serve as a foundation for future structural exploration of the ligase in the larger context of the NHEJ complex.

## Methods

### Expression and purification of human LigIV catalytic domain

The pOPINS-hLigIV vector encoding residues Met1-Asp620 of human LigIV, fused downstream of a 6xHis-SUMO tag^20^ was a kind gift from T. Blundell. The vector was transformed into Rosetta2 (DE3) pLysS cells (EMD Millipore) for expression. The cells were grown at 37 °C with shaking at 275 rpm in Terrific broth containing 50 μg mL^−1^ kanamycin and 35 μg mL^−1^ chloramphenicol, to an OD_600nm_ of 0.9. At this point, the temperature was decreased to 16 °C for 40 min. Protein expression was induced by addition of IPTG to a final concentration of 1 mM, and proceeded overnight at 16 °C. The cells were lysed by sonication in Buffer A (25 mM Tris pH 8, 1 M NaCl, 10% glycerol, 1 mM β-mercaptoethanol (βME), 10 mM imidazole), in the presence of 1 mM PMSF and cOmplete Protease Inhibitor Cocktail (Roche). Polyethyleneimine was added dropwise to the lysate to a final concentration of 0.1%, and clarified by centrifugation. The resulting supernatant was bound in-batch to Ni-NTA resin (Qiagen) for 1 h at 4 °C. Bound protein was eluted using a linear gradient to 100% Buffer B (25 mM Tris pH 8, 1 M NaCl, 10 % (v/v) glycerol, 1 mM βME, 300 mM imidazole). Pooled fractions were mixed with 1 mg of Ulp1 SUMO-specific protease, and dialyzed overnight against Buffer C (25 mM Tris pH 8, 500 mM NaCl, 5% (v/v) glycerol, 2 mM βME) at 4 °C. The dialyzed protein was concentrated and loaded onto a Superdex 200 26/60 size exclusion column (GE Healthcare). Pooled fractions were dialyzed overnight against Buffer D (25 mM Tris pH 8, 100 mM NaCl, 10 % (v/v) glycerol, 2 mM βME) at 4 °C. The dialyzed protein was loaded onto a MonoQ HR 5/5 column equilibrated in Buffer D, and the LigIV catalytic domain was observed in the flowthrough. This protein was then bound to a HiTrap Heparin HP column equilibrated in Buffer D, and eluted with a linear gradient to 100% Buffer C. Pooled fractions were dialyzed overnight at 4 °C against a final storage buffer comprised of 25 mM Tris pH 8, 100 mM NaCl, 5 % (v/v) glycerol, 1 mM dithiothreitol, and concentrated to ~12 mg mL^−1^. All proteins were flash-frozen in liquid nitrogen and stored at −80 °C.

### Co-crystallization of human LigIV with nicked DNA substrate

The following DNA oligonucleotides were used to create the nicked DNA complex: template (5′-GTCCGACGACGCATCAGC-3′), dideoxy-terminated upstream primer (5′-GCTGATGCGTddC-3′), and a 5′-phosphorylated downstream primer (5′-pGTCGGAC-3′). These oligos were resuspended in ddH_2_O, mixed in equimolar ratios, and annealed in a thermal cycler by denaturation at 94 °C, followed by a slow temperature gradient from 90 °C to 4 °C. Due to the poor extent of LigIV adenylation in vivo, the protein was pre-incubated with 4 mM MgCl_2_ and 1 mM ATP for 1 h on ice. The annealed DNA was then added at a 2:1 molar ratio, and allowed to bind for 1 h on ice at 4 °C. Crystals of the DNA–adenylate were grown at room temperature, by mixing 300 nL of the protein:DNA complex with 300 nL mother liquor (78.7 mM HEPES pH 7.5, 7.87 % (v/v) isopropanol, 15.64 % (w/v) PEG4000, 13.8 % (v/v) glycerol), using the sitting-drop vapor diffusion technique^43^. Crystals of the lysyl–adenylate were also grown by sitting-drop vapor diffusion at room temperature, by mixing 300 nL of the protein:DNA complex with 300 nL of mother liquor (77 mM HEPES pH 7.5, 7.7% (v/v) isopropanol, 15.3% (w/v) PEG4000, 13.5% (v/v) glycerol, 10 mM MgCl_2_). For cryopreservation, 1 μL of a cryopreservation solution (77 mM HEPES pH 7.5, 7.7% (v/v) isopropanol, 15.3% (w/v) PEG4000, 18.5% (v/v) glycerol, 10 mM MgCl_2_ for the lysyl–adenylate crystals and 81.2 mM HEPES pH 7.5, 8.12% (v/v) isopropanol, 16.2% (w/v) PEG4000, 19.3% (v/v) glycerol for the DNA–adenylate crystals) was added to the original crystal growth drop. The crystals were then flash-frozen in liquid nitrogen and placed into a stream of nitrogen gas cooled to –173 ° C for data collection.

### Structure solution and refinement

The data were collected at a wavelength of 1 Å on the Southeast Regional Collaborative Access Team (SER-CAT) 22-ID beamline at the Advanced Photon Source at Argonne National Laboratory, with a Rayonix MX300HS detector. The data were indexed, integrated, and scaled using HKL2000^44^. The phase problem for the DNA–adenylate complex was solved by molecular replacement with Phaser^45^, using residues Gln8-Glu453 of the DBD and NTD subdomains of the LigIV apoprotein structure (PDB ID code 3W5O^20^) as the search model. A second round of molecular replacement using this model as a fixed partial solution was required to determine the location of the OBD subdomain, using residues Glu461-Arg603 of PDB ID code 3W5O as the search model. The phase problem for the lysyl–adenylate complex was solved by molecular replacement with Phaser, using the entire LigIV apoprotein structure (PDB ID code 3W5O^20^) as a search model. All structures were refined using iterative cycles of manual model building in COOT^46,47^ and Phenix^48^. TLS refinement was used for both structures, and refinement of the lysyl–adenylate complex included Ramachandran restraints. Data refinement statistics are listed in Supplementary Table 1. Ramachandran statistics were determined using MolProbity^49^—the DNA–adenylate and protein–adenylate complexes contained 96.6% and 96.4% of all residues in favored regions, respectively. All structural figures were generated using the PyMOL Molecular Graphics System, Version 1.5 (Schrödinger, LLC).

### Generation of human LigIV substitution mutants

The 6xHis-tag on the original SUMO fusion of the wild-type protein was augmented to include ten histidine residues for improved binding to Ni-NTA resin. Amino-acid substitution mutants were subsequently generated on this background using site-directed mutagenesis. Primer sequences used for mutagenesis are shown in Supplementary Table 5 The mutants were expressed and lysed in a similar fashion as for the wild-type protein. For their purification, soluble mutant proteins were bound in-batch to Ni-NTA resin and loaded onto a gravity flow column. Unbound proteins were removed by washing with Buffer A containing an additional 5 mM imidazole, and the bound protein was collected using batch elution with Buffer B. Subsequent purification steps were performed identically to that of the wild-type protein. All mutants behaved indistinguishably from wild type during size exclusion chromatography.

### DNA ligation activity assays

DNA substrates for ligation assays were prepared by hybridizing an upstream primer (5′-GTCACCTGATGCGTC-3′) and a 5′-phosphorylated, 3′-Cyanine3-labeled downstream primer (5′-pGTCGGACTACTGAGT-Cy3-3′ for unadenylated substrate L4 and 5′-AMP-pGTCGGACTACTGAGT-Cy3-3′ for pre-adenylated substrate L4A) to a dideoxycytidine-terminated template primer (5′-ACTCAGTAGTCCGACGACGCATCAGGTGAddC-3′) to create a nicked DNA duplex. Reaction mixtures (20 µL) contained 50 mM Tris, pH 8, 1 mM DTT, 4 % (v/v) glycerol, 0.1 mg mL^−1^ BSA, 50 nM DNA substrate, 5 mM MgCl_2_. 1 mM ATP was added to aid in enzyme adenylation on the unadenylated DNA substrate, where indicated. Reactions were initiated by adding the catalytic domain construct (residues Met1-Asp620) of LigIV (WT or mutant) at 500 nM and incubating at 37 °C for 1 h. The reactions were quenched by addition of an equal volume of loading dye (99 % (v/v) formamide, 10 mM EDTA, 0.1% (w/v) xylene cyanol, and 0.1% (w/v) bromophenol blue). The products were resolved on a 16 % denaturing polyacrylamide gel, imaged using a Typhoon 9400 imager (GE Healthcare Life Sciences) and quantified using ImageQuant software. Statistical significance of ligation product formation between WT and LigIV variants was assessed using the two-tailed Student’s *t*-test of three independent replicates.

### Data availability

Atomic coordinates and structure factors have been deposited in the Protein Data Bank (www.pdb.org) with ID codes 6BKF and 6BKG. All other data supporting the findings of this study are available within the article and its Supplementary Information file, or are available from the authors upon reasonable request.

## Electronic supplementary material

Supplementary Information
