# Supplementary material for: Structures of DNA-bound human ligase IV catalytic core reveal insights into substrate binding and catalysis
Source: Nat Commun. 2018 Jul 6;9:2642. doi: 10.1038/s41467-018-05024-8 (PMC6035275; doi:10.1038/s41467-018-05024-8)
Supplement: Supplementary file 1 — Supplementary Information [file 41467_2018_5024_MOESM1_ESM.pdf]

Structures of DNA-bound human Ligase IV catalytic core reveal insights into substrate binding and catalysis

Kaminski *et al*

SUPPLEMENTARY FIGURES

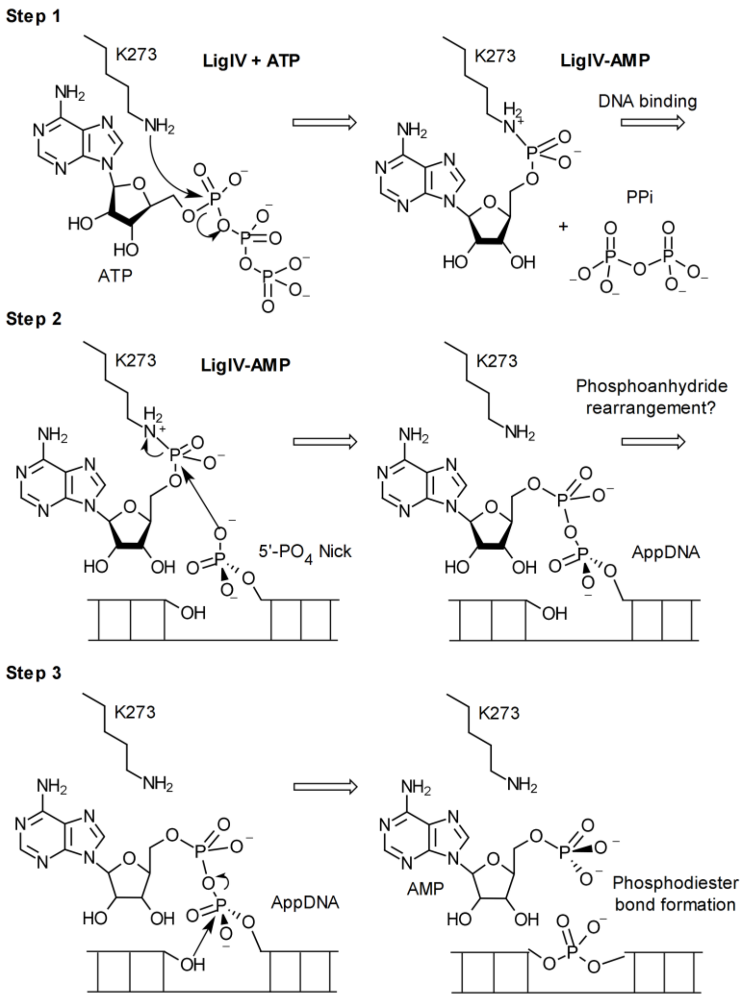

Supplementary Figure 1: Proposed reaction mechanism for ligation. Step 1 (top) refers to the auto-adenylation reaction of LigIV, involving an attack by an active site lysine (Lys273) on the α-phosphate of a bound ATP cofactor, which results in formation of a covalent lysyl-adenylate intermediate. Step 2 (middle) illustrates binding of the lysyl-adenylate complex of LigIV to a nicked DNA substrate, as captured in PDB ID code 6BKF. The adenylate group is transferred to the 5'-phosphate of the downstream strand in the nicked complex, as captured in PDB ID code 6BKG. The nick is sealed in Step 3 (bottom), when the 3'-OH (not present in the reported structures) on the upstream primer terminus attacks the 5'-phosphorus atom on the downstream strand, forming a new phosphodiester bond and releasing AMP.

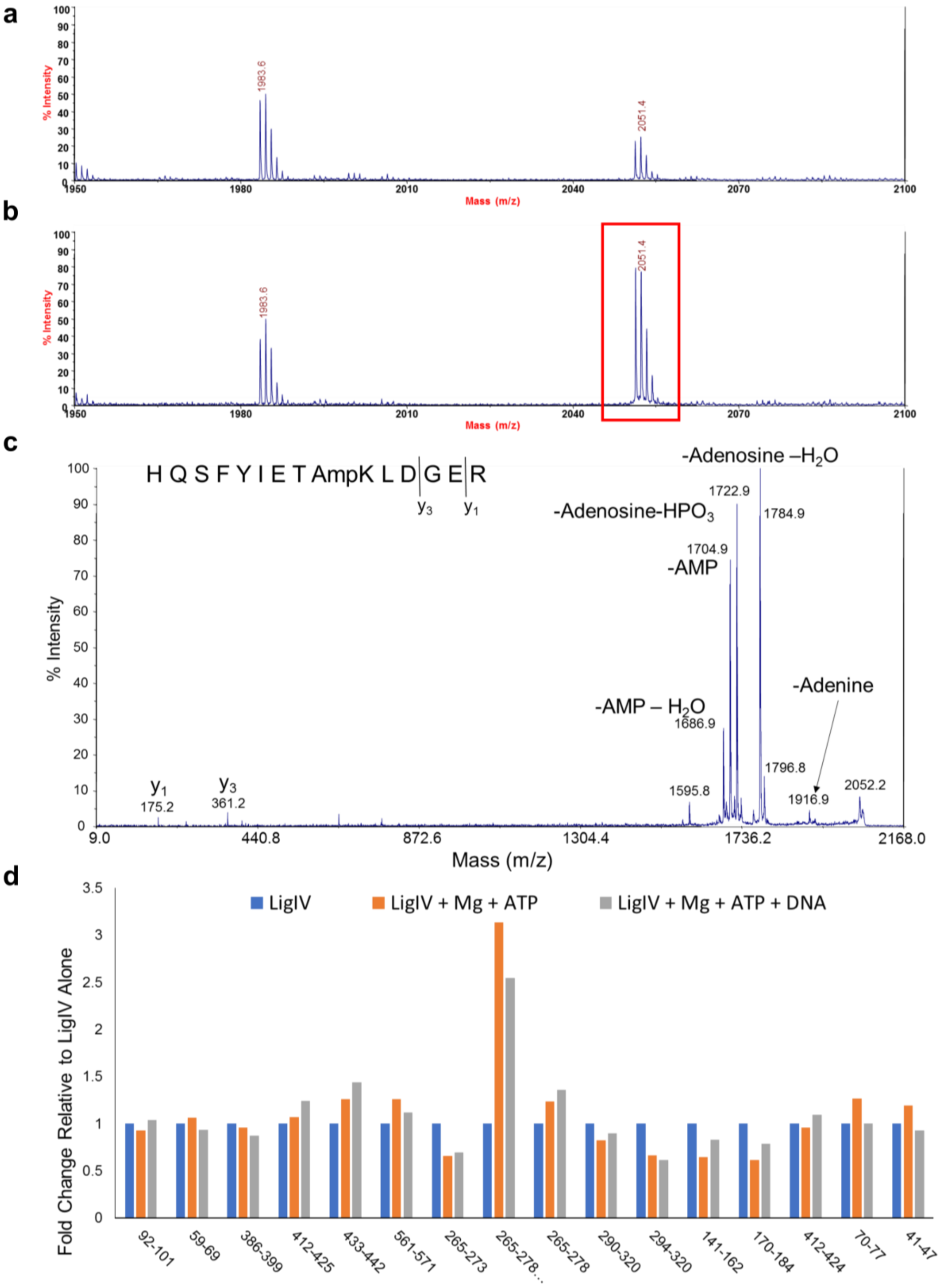

Supplementary Figure 2: Assessment of LigIV catalytic domain adenylation by mass spectrometry. **a.** Purified recombinant LigIV catalytic domain (residues Met1-Asp620) is poorly adenylated on Lys273, as

indicated by the low intensity ion at m/z 2051.4, which matches the predicted mass of the tryptic peptide spanning from His265-Arg278, with adenylation at Lys273. **b.** The extent of adenylation could be increased by pre-incubating with ATP and $MgCl_2$ prior to co-crystallization with nicked DNA substrate, as indicated by the increased intensity of the ion at m/z 2051.4 (red box). **c.** MS/MS of the ion m/z 2051.4 confirms its identity as HQSFYIET(Amp)KLDGER with multiple fragmentation events of the adenylate moiety and a limited y-ion series. **d.** MALDI-MS spectra were collected for tryptic digests of purified LigIV catalytic domain (blue), LigIV pre-incubated with ATP and $MgCl_2$ (orange), and LigIV incubated with ATP, $MgCl_2$, and nicked DNA prior to crystallization (gray). The intensities of all ions that were readily assigned to tryptic peptides of LigIV were normalized relative to the average of the intensity of the 4 most abundant ions in each spectrum (m/z 1243.4 → residues Leu92-Arg101; m/z 1301.3 → residues Asp59-Arg69; m/z 1592.5 → residues Tyr386-Leu389; m/z 1599.4 → residues Asn412-Arg425). Ratios of these normalized values were then taken relative to the normalized abundance for LigIV alone.

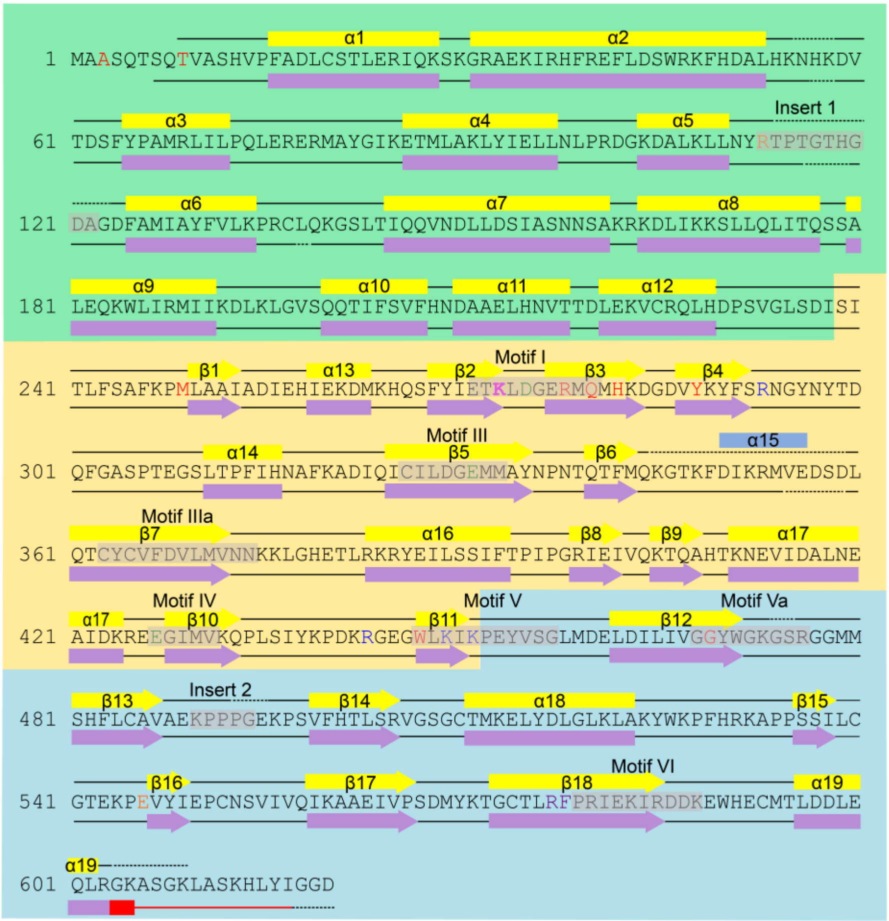

Supplementary Figure 3: Sequence and secondary structural elements of the human LigIV catalytic domain. Residues comprising the DBD, NTD, and OBD subdomains are boxed in light green, orange, and blue, respectively. The sequence is shown, with the corresponding secondary structural elements of the DNA-bound protein-adenylate (yellow) or DNA-adenylate (purple) complexes, with the α-helices and β-strands portrayed as rectangles or arrows, respectively. Disordered regions are indicated by dashed lines. Lys273, the site of adenylation, is colored magenta. α-helix 15 (blue rectangle), which was ordered in the apoprotein structure (PDB ID code 3W5O[1]), becomes disordered when DNA is bound. α19 (red)—disordered in the apoprotein and lysyl-adenylate DNA-bound complex—is extended in the closed conformation of the DNA-adenylate complex, and the C-terminal tail not included in the crystallized construct of the apoprotein[1] becomes ordered (red). Residues that may be involved in metal binding, charge neutralization, 'latch' mechanism, or stabilization of primer terminal base positioning are colored in

green, blue, orange, and violet, respectively. Mutations that have been associated with LIG4 Syndrome are colored in red.

a

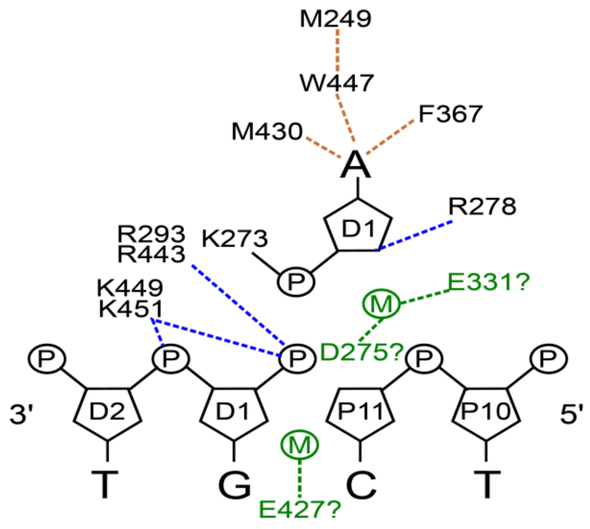

b

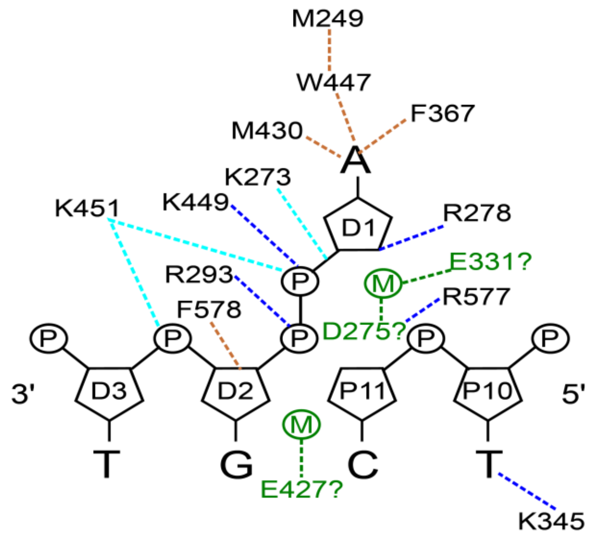

Supplementary Figure 4: Interaction map for LigIV catalytic domain engaging a nicked DNA substrate in either the open lysyl-adenylated conformation (**a**) or in the closed DNA-adenylate form (**b**). Interactions between the DNA and protein side chains are indicated as dashed lines (hydrogen bonds in blue, putative long-range hydrogen bonds in cyan, potential metal interactions in green, and van der Waals interactions in brown).

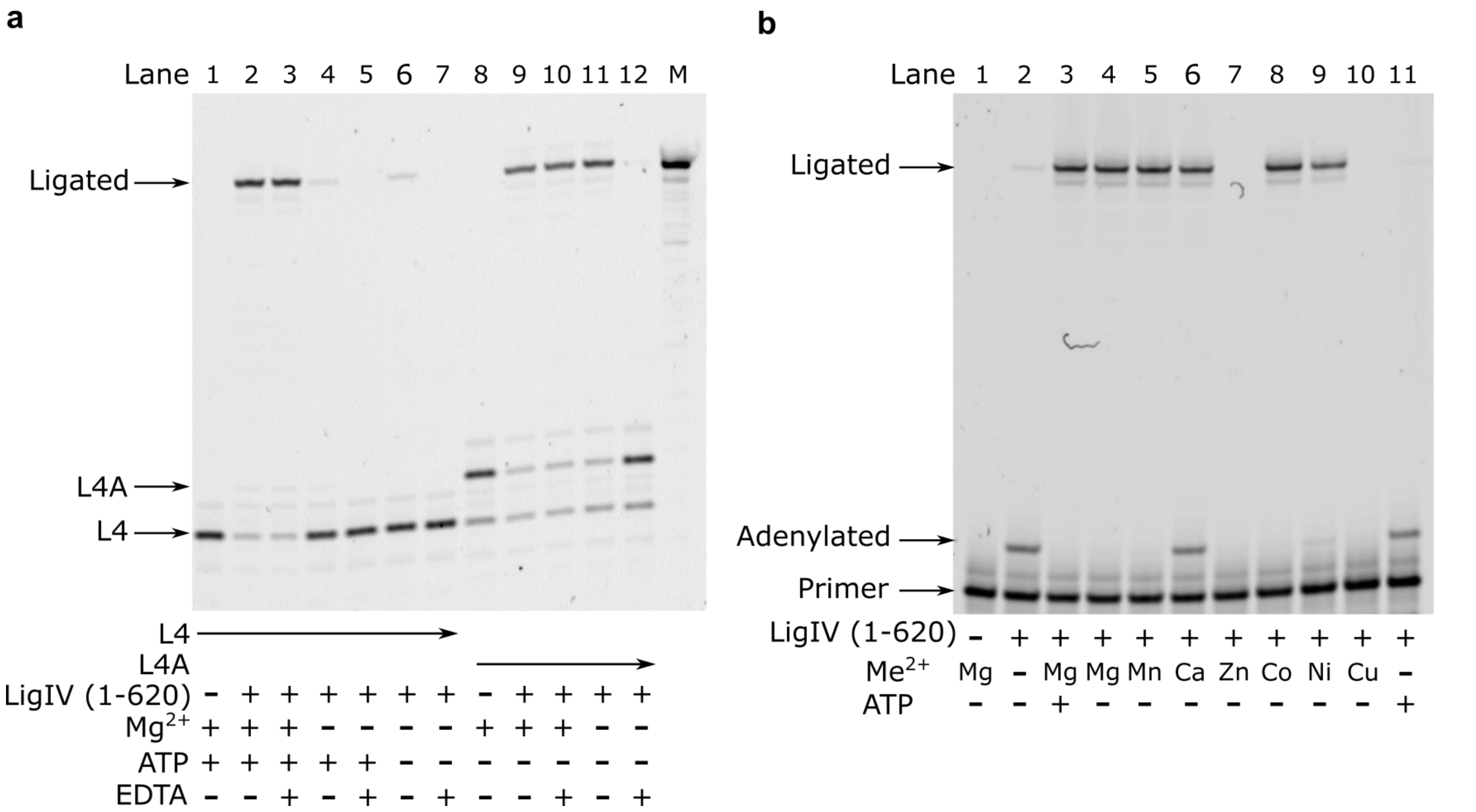

Supplementary Figure 5a-5b

c

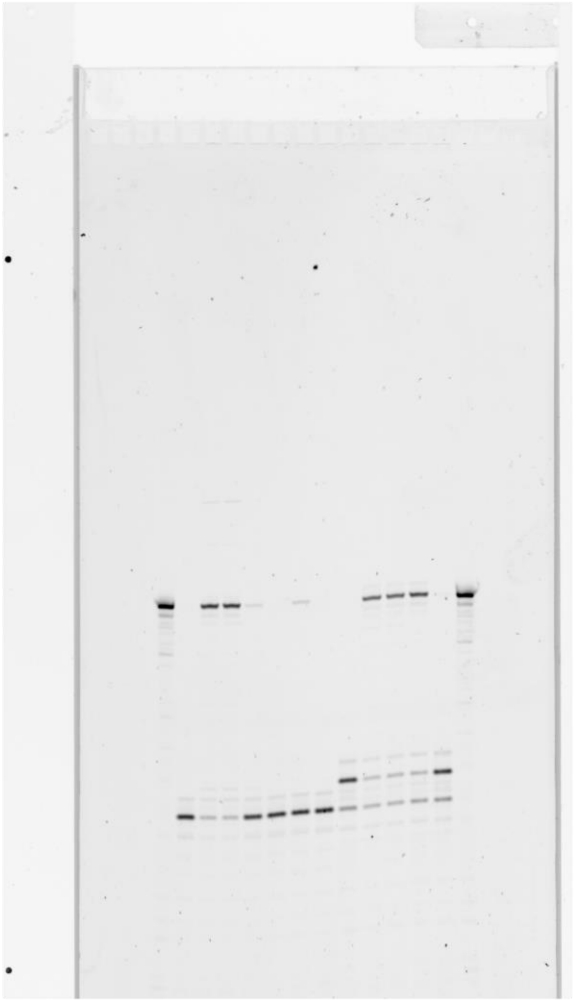

d

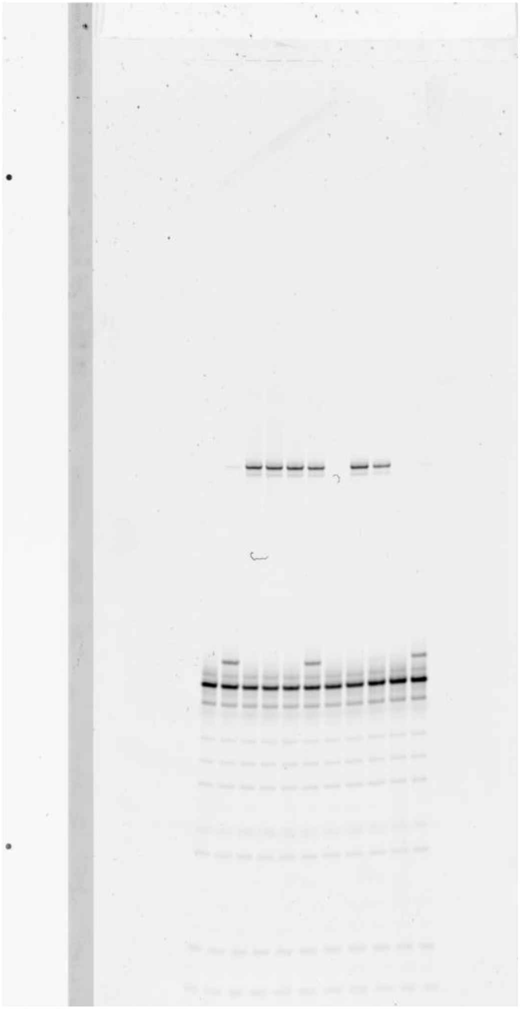

Supplementary Figure 5c-5d

Supplementary Figure 5: Assessment of divalent metal-dependence of LigIV. **a.** The LigIV catalytic domain (500 nM) was tested for ligation activity on either the L4 unadenylated or L4A pre-adenylated nicked DNA substrate (50 nM). Reactions were carried out at 37° C for 1 hour in the presence or absence of 5 mM $MgCl_2$, 1 mM ATP, or 2 mM EDTA, as indicated. The EDTA concentration (2 mM) was chosen to ostensibly chelate any trace levels of divalent metals carried through from the purification, while permitting ligation in excess of exogenously added Mg^{2+} (5 mM added, likely 1 mM after EDTA chelation). The small amount of ligation products formed in lanes 4, 6, and the robust ligation product formation in lane 11, in the absence of 2 mM EDTA, suggest the presence of trace amounts of divalent metals present, carried through from the purification of the LigIV catalytic domain. **b.** Visualization of nick ligation products generated by the LigIV catalytic domain (500 nM), using an unadenylated nicked DNA substrate (50 nM), in the presence of 5 mM divalent metal ions and 1 mM ATP (as indicated), after a 3 minute incubation at 37° C. Accumulation of adenylated primer, and small amount of ligation products formed in lanes 2 and 11 suggest the presence of trace amounts of divalent metals present in the protein carried through the purification process. That these products represent only a fraction of converted primer is indicative of the poor extent of adenylation of the LigIV catalytic domain construct. All ligation products were visualized using denaturing polyacrylamide gel electrophoresis, using a Typhoon 9400 imager. Uncropped gel images for panels **a** and **b** are shown in panels **c** and **d**, respectively.

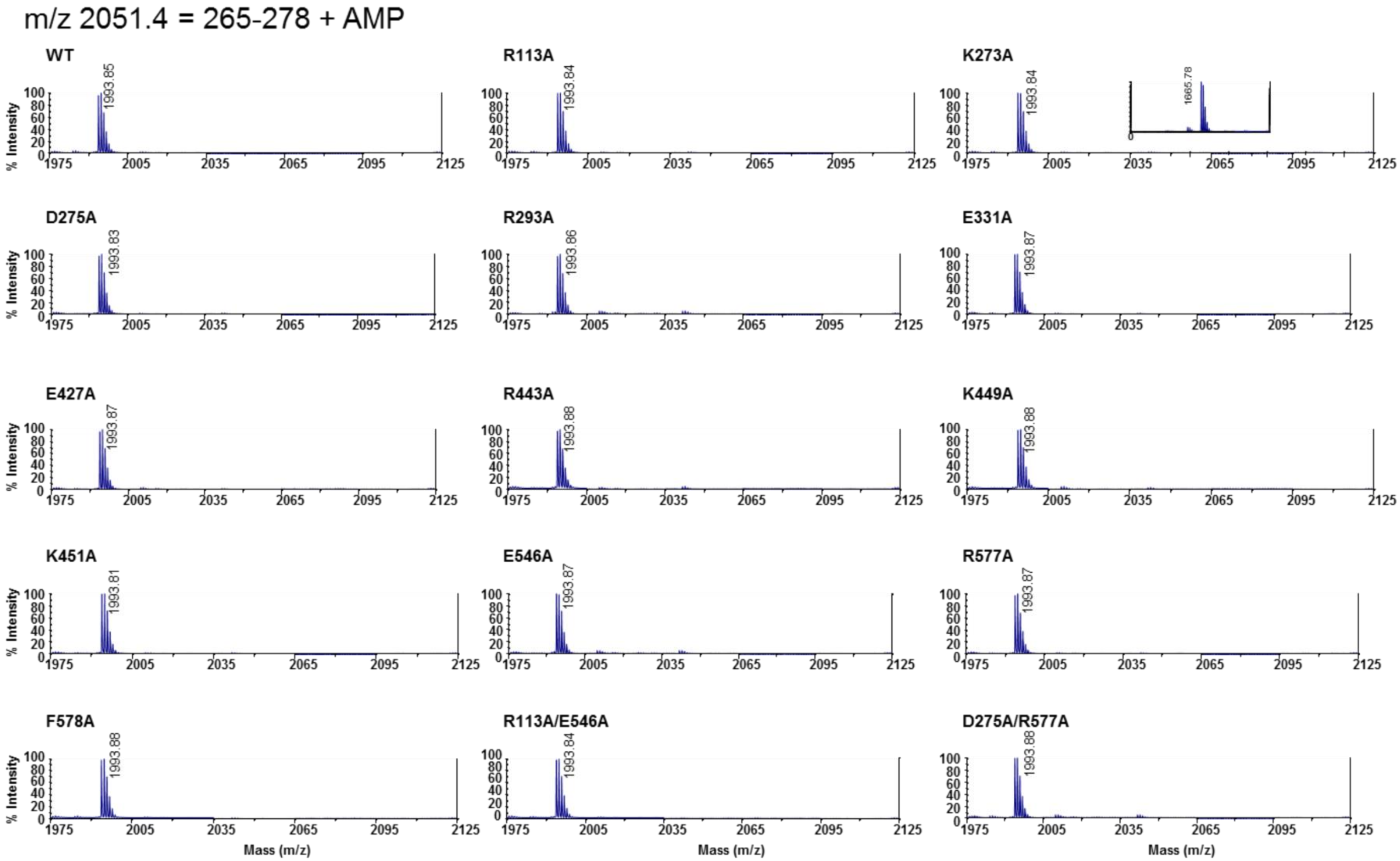

Supplementary Figure 6a

m/z 1152.57 = His265-Lys273

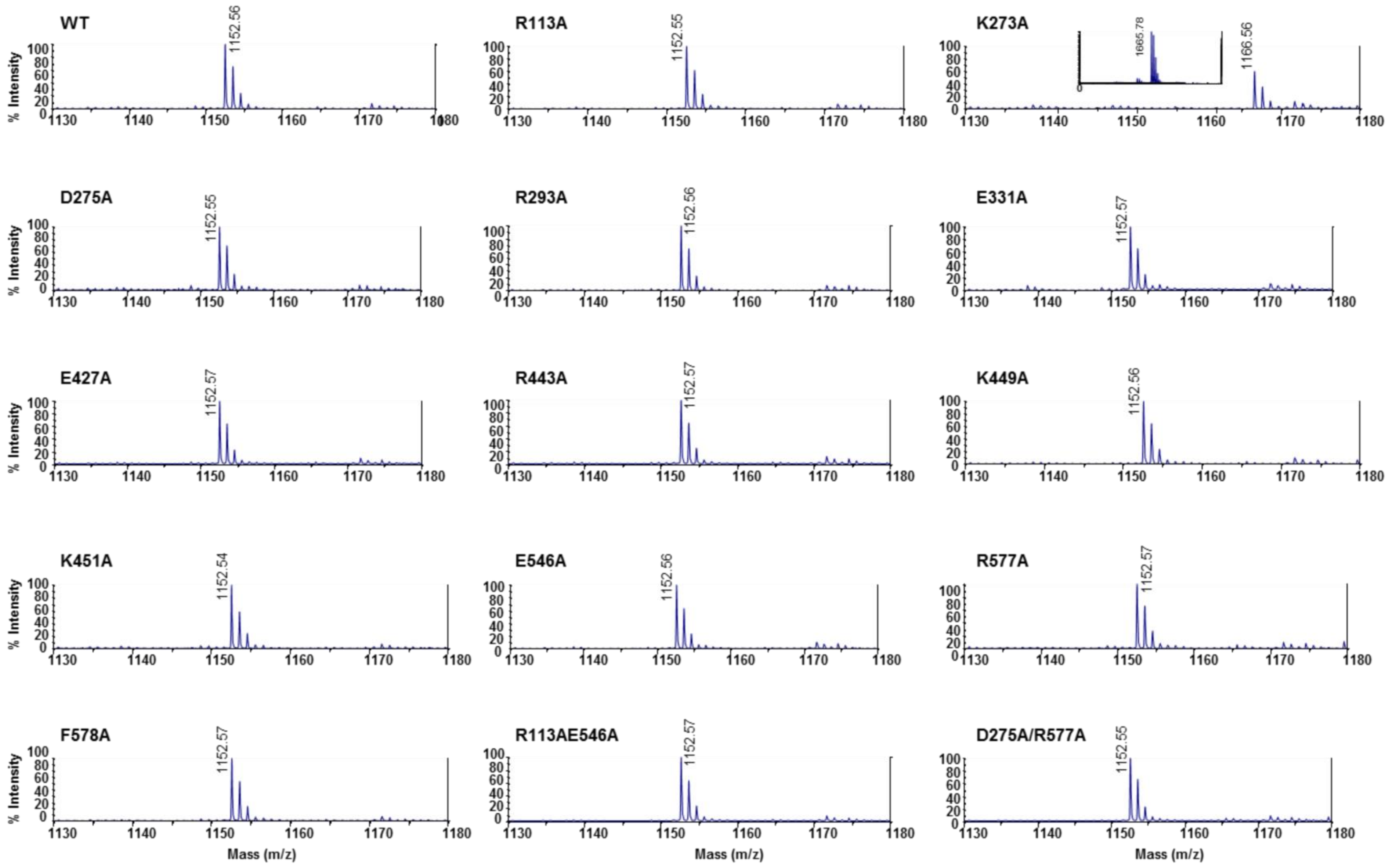

Supplementary Figure 6b

Supplementary Figure 6: Assessment of the extent of *in vivo* adenylation of the LigIV catalytic domain mutants by mass spectrometry. **a.** MS spectra of region spanning m/z 1975 to m/z 2125. Wildtype and mutant LigIV catalytic domain constructs were analyzed by MALDI mass spectrometry, for the presence of the Lys273 lysyl-adenylate peptide (m/z 2051.4) as shown in **Suppl. Fig. 2a-b**. The K273A mutant cannot be adenylated (or cleaved by trypsin) at Ala273 and the resulting tryptic peptide (m/z 1665.78) is displayed as an inset. **b.** MS spectra of region spanning m/z 1130 to m/z 1180. Wildtype and mutant LigIV catalytic domain constructs were analyzed by MALDI mass spectrometry, for the presence of the peptide spanning from His265 to Lys273 (m/z 1152.6). In the absence of adenylation, Lys273 is readily recognized and hydrolyzed by trypsin. Adenylation of this series of LigIV mutants was not readily detected. The Lys273 to Ala mutant cannot be adenylated (or cleaved by trypsin) at Ala273 and the resulting tryptic peptide (m/z 1665.78) is displayed as an inset.

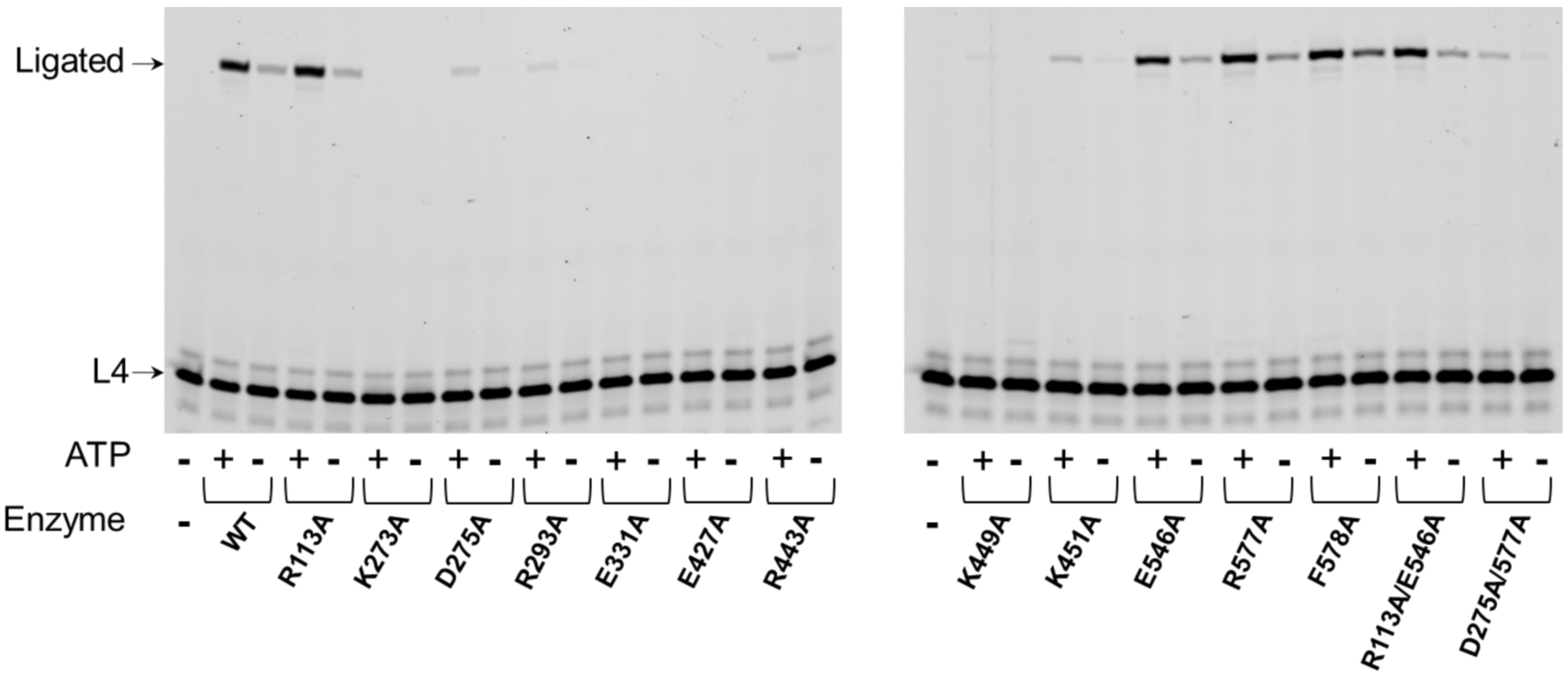

Supplementary Figure 7a

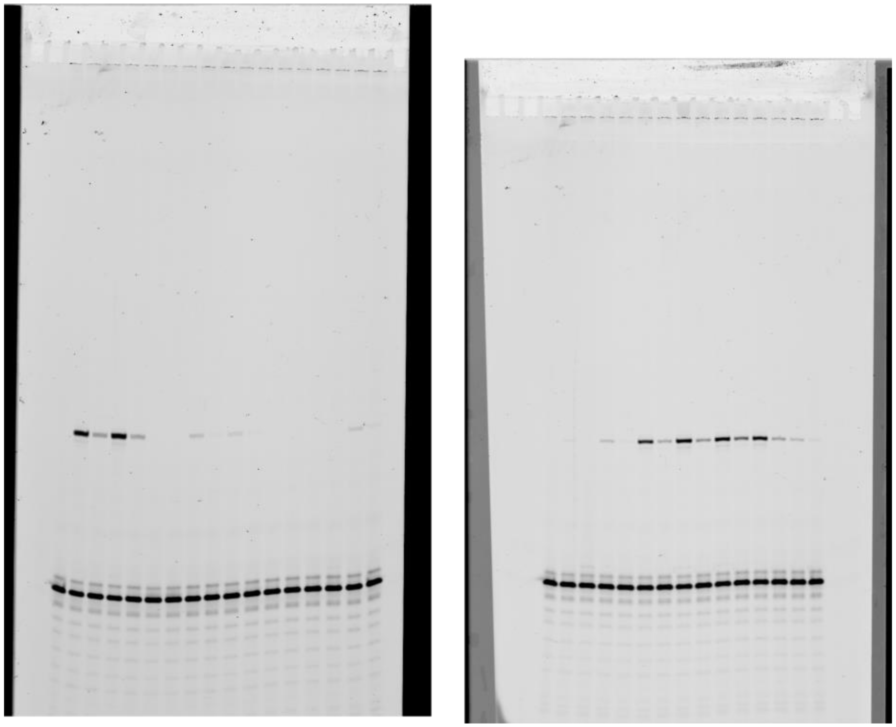

Supplementary Figure 7b

Supplementary Figure 7: Visualization of nick ligation products by the wildtype and mutant LigIV catalytic domains. **a**. Purified recombinant catalytic domain variants were incubated with a nicked DNA substrate (L4) of identical sequence context to that used in crystallization, in the presence of 5 mM $MgCl_2$, with or without 1 mM ATP (as indicated). The resulting Cy3-labeled ligation products were visualized using denaturing polyacrylamide gel electrophoresis, and quantitated using a Typhoon 9400 imager and ImageQuant software. Uncropped images of gels in panel **a** are shown in panel **b.**

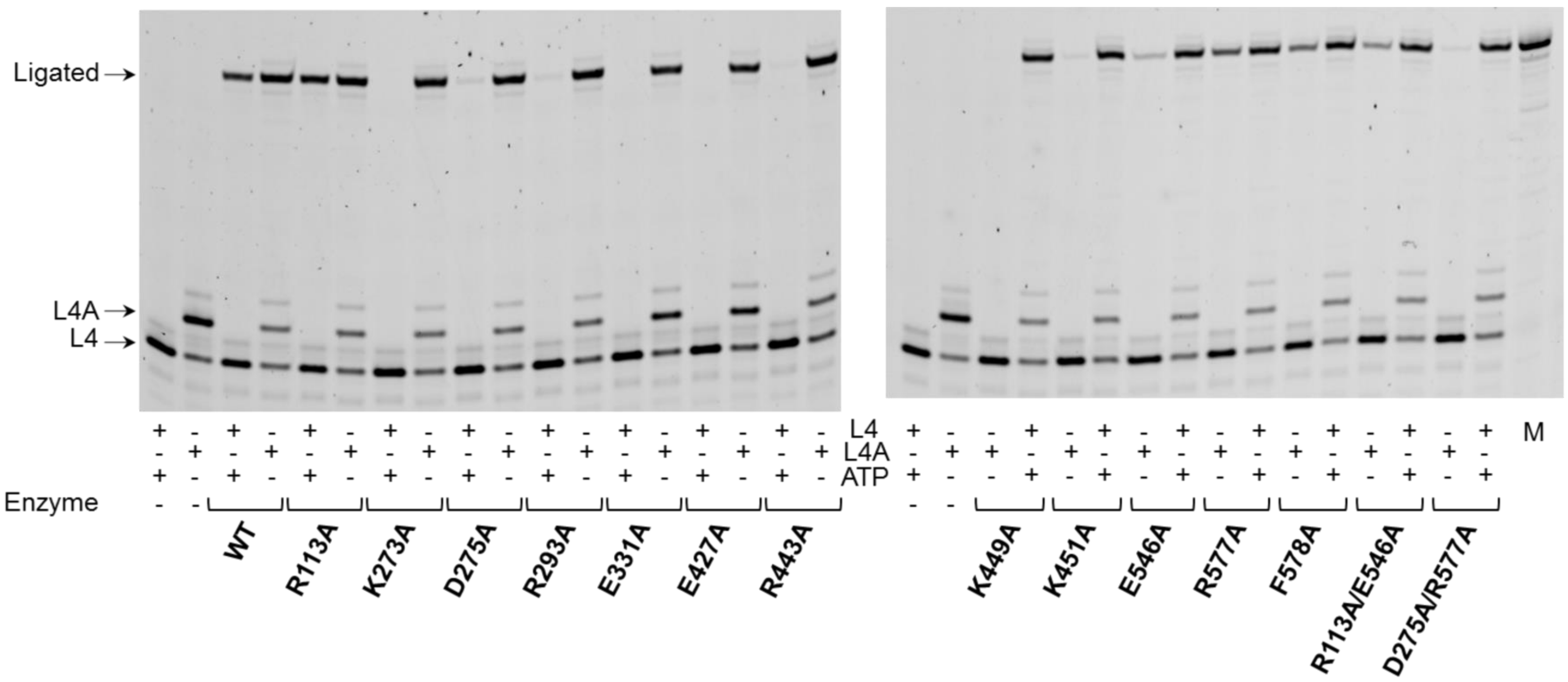

Supplementary Figure 8a

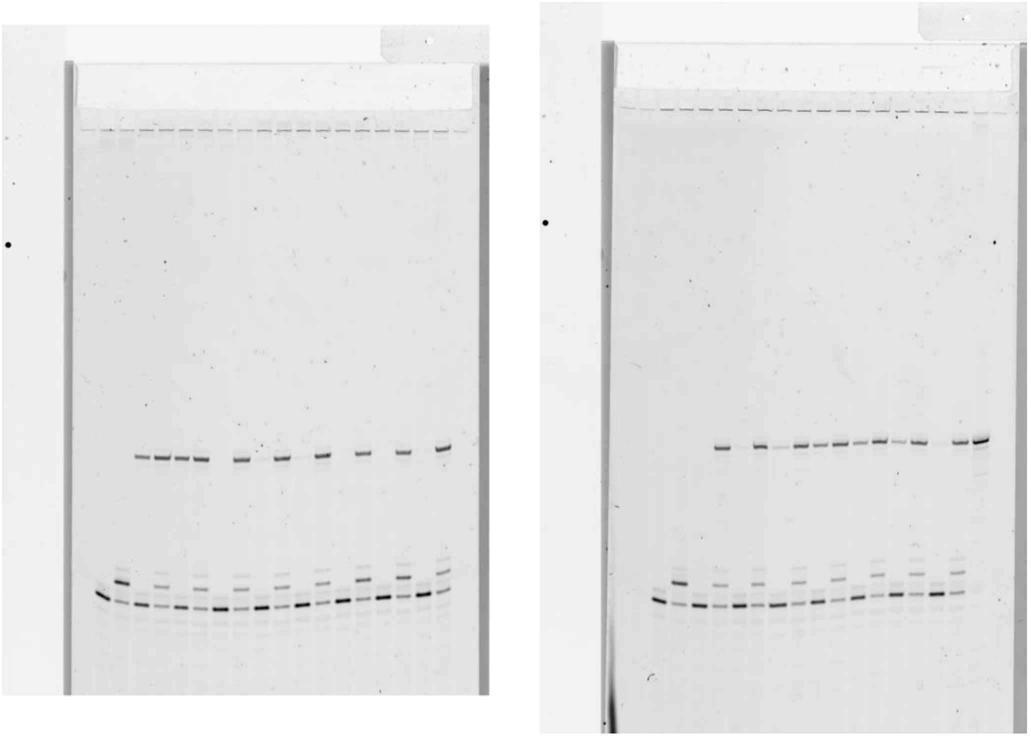

Supplementary Figure 8b

Supplementary Figure 8: Visualization of nick ligation products by the wildtype and LigIV catalytic domain mutants. **a**. Ligation activity assays were performed using either an unadenylated (L4, with 1 mM ATP) or pre-adenylated (L4A, without ATP) substrate. The resulting Cy3-labeled ligation products were visualized using denaturing polyacrylamide gel electrophoresis, and quantitated using a Typhoon 9400 imager and ImageQuant software. The lane marked 'M' contains a 3'-end Cy3-labelled 30mer representing the ligated product. Uncropped images of gels in panel **a** are shown in panel **b**.

SUPPLEMENTARY TABLES

Supplementary Table 1: Data collection and refinement statistics

	Lig IV with bound DNA (lysyl-adenylate)[a,b]	Lig IV with bound DNA (DNA-adenylate)[a,b]
PDB ID code	6BKF	6BKG
**Data collection**		
Space group	$P4_32_12$	$P2_12_12_1$
Cell dimensions		
a, *b*, *c* (Å)	199.22, 199.22, 137.41	72.66, 102.75, 110.13
α, β, γ (°)	90, 90, 90	90, 90, 90
Resolution (Å)	50-3.25 (3.31-3.25)[c]	50-2.40 (2.44-2.40)
R_{sym} (%)	7.9 (38.9)	6.5 (84.6)
$I / \sigma I$	20.04 (3.00)	31.16 (2.45)
Completeness (%)	98.0 (98.8)	100 (100)
Redundancy	5.2 (5.5)	7.3 (7.4)
**Refinement**		
Resolution (Å)	37.70-3.25	39.20-2.40
No. reflections	14106	32630
R_{work} / R_{free} (%)	26.38/31.57	20.14/24.50
No. atoms		
Protein	4104	4625
DNA	732	742
AMP	22	29
Water	3	112
B-factors		
Protein	69.28	43.17
DNA	75.18	41.65
AMP	45.69	41.64
Water	36.74	38.08
R.m.s. deviations		
Bond lengths (Å)	0.006	0.003
Bond angles (°)	0.988	0.585

[a]A single crystal was used to collect each data set

[b]These crystals were collected on the Southeast Regional Collaborative Access Team (SER-CAT) 22-ID beamline at the Advanced Photon Source at Argonne National Laboratory.

[c]Values in parentheses are for highest-resolution shell.

Supplementary Table 2: Putative hydrogen bonding interactions with ligands in the LigIV catalytic core complexes

Complex	LigIV residue/atom	Ligand residue/atom	Distance (Å)
Lysyl-adenylate[a] (open conformation)	Arg278 NH1	AMP O2′	2.9
	Arg293 NH1	D2 OP2	2.8
	Arg293 NH2	D2 OP1	2.7
	Arg443 NH1	D2 OP1	2.9
	Lys449 NZ	D2 OP3	3.3
	Lys449 NZ	D3 OP2	3.3
	Lys451 NZ D3	D3 OP1	2.8
	AMP O3′	D2 OP3	2.5
DNA-adenylate[b] (closed conformation)	Lys273 NZ	AMP O5′	3.1
	Lys273 NZ	AMP O2P	3.3
	Arg278 NH1	AMP O3′	3.0
	Arg293 NH1	D2 OP1 (A)[c]	3.2
	Arg293 NH1	D2 OP1 (B)[c]	3.0
	Lys345 NZ	P10 O2	2.6
	Lys348 N	T11 OP1	3.3
	Lys449 NZ	AMP O1P	2.4
	Lys451 NZ	D2 O3′	3.2
	Lys451 NZ	D3 OP1	3.2
	Lys451 NZ	AMP O2P	3.4

[a]The lysyl-adenylate complex was solved at 3.25 Å resolution, with a maximum-likelihood based estimated coordinate error of 0.44 Å.
[b]The DNA-adenylate complex was solved at 2.4 Å resolution, with a maximum-likelihood based estimated coordinate error of 0.3 Å.
[c]The A and B designations correspond to the respective alternate conformations of the phosphoanhydride linkage modeled in this structure.

Supplementary Table 3: Interactions observed at the DBD/OBD interface in the DNA-adenylate closed conformation

	DBD residue	OBD residue	Distance (Å)
**Hydrogen bonds**	Arg113 NH1	Glu546 OE2	3.0
	Arg113 NH2	Glu546 OE2	2.8
	Arg113 NH1	Trp471 O	2.7
	Gly123 O	Gly474 N	3.0
**van der Waals**	Tyr112	Lys473	3.4
	Arg113	Trp471	3.3-4.0[a]
	Arg113	Gly472	3.3-3.7[a]
	Arg113	Lys473	3.9
	Thr114	Trp471	3.6
	Pro115	Trp471	3.7-3.8[a]
	Ala122	Gly477	3.3-3.6[a]
	Gly123	Lys473	3.8

[a]Distance ranges are given when multiple atoms between residues are found to be within van der Waals interaction distance. Interaction distances for this interface were calculated using CNS[2].

Supplementary Table 4: Structure-based sequence alignment of LigIV regions of interest with those of similar ligases

Ligase	Insert 1	Motif 1	R293A	Motif III	Motif IV
hLigIV	LLNYRTPT (R113)	YIETKLDGERM (K273/D275)	KYFSRNGYN (R293)	ILDGEMMAY (E331)	DKREEGIMV (E427)
hLigI	VRAEAEKG (~A374[a])	TCEYKYDGQRA (K568/D570)	KIFSRNQED (R589)	ILDTEAVAW (E621)	KDSCEGLMV (E720)
hLigIII	ARCLEQGD (~E259[a])	FSEIKYDGERV (K421/D423)	SYFSRSLKP (R441)	ILDSEVLLI (E473)	QEGLEGLVL (E568)
Chlorella	b	LATPKIDGIRS (K27/D29)	QMLSRTFKP (R42)	GSDGEISIE (E67)	SKGFEGVMI (E161)
T4 Rnl1	b	YILTKEDGSLV (K99/D101)	LFKSKGSIK (K119)	TANFEFVAP (E159)	AENIEGYVA (E227)
T4 Rnl2	b	VAREKIHGTNF (K35/H37)	TCAKRTGPI (R55)	QVFGEFAGP (E99)	VFTAEGYVL (E204)

Ligase	R443 and Motif V	E546A	R577A/F578A
hLigIV	KPDKRGEG----WLKIKPEYV (R443/K449/K451)	TEKPEVYIE (E546)	GCTLRFPRIE (R577/F578)
hLigI	AKRSHN------WLKLKKDYL (H740[c]/K744/K746)	AVIPDHWLD (~D833[a])	GISLRFPRFI (R871/F872)
hLigIII	EPGKRH------WLKVKKDYL (R584[c]/K588/K590)	b	GISIRFPRCT (R716/F717)
Chlorella	YKFGRSTLKEGILLKMKQFKD (R176[c]/K186/K188)	b	KDCPRFPVFI (R285/F286)
T4 Rnl1	....K.....GSHFKIKSDWY (~K75[d]/K240/K242)	b	b
T4 Rnl2	..........RVAIKCKNSKF ([e]K225/K227)	b	b

[a]The DBD/OBD interface in the closed ligase structures are not well conserved, so the indicated residues represent roughly equivalent structural positions.
[b]Regions with no structural homology.
[c]The β10-β11 connecting loop of LigIV is of varying length and structure in these ligases.
[d]The global structure and connectivity of T4 Rnl1 is entirely dissimilar in this vicinity, but K75 lies in the equivalent position of Arg443 in LigIV.
[e]The global structure and connectivity of T4 Rnl2 is entirely dissimilar in this vicinity, and has no structural equivalent for Arg443.

Supplementary Table 5: Primer sequences used in site-directed mutagenesis

Mutant	Primer Sequences[1]
R113A	Forward: GCCCTCAAACTTTTAAACTACGCAACACCCACTGGAACTCATGG<br>Reverse: CCATGAGTTCCAGTGGGTGTTGCGTAGTTTAAAAGTTTGAGGGC
K273A	Forward: AGAGTTTCTACATAGAAACCGCGCTAGATGGTGAACGTATGC<br>Reverse: GCATACGTTCACCATCTAGCGCGGTTTCTATGTAGAAACTCT
D175A	Forward: CATAGAAACCAAGCTAGCTGGTGAACGTATGCAAATGC<br>Reverse: GCATTTGCATACGTTCACCAGCTAGCTTGGTTTCTATG
R293A	Forward: GGAGATGTATATAAATACTTCTCTGCAAATGGATATAACTACACTG<br>Reverse: CAGTGTAGTTATATCCATTTGCAGAGAAGTATTTATATACATCTCC
E331A	Forward: CTGTATTCTTGATGGTGCGATGATGGCCTATAATCC<br>Reverse: GGATTATAGGCCATCATCGCACCATCAAGAATACAG
E427A	Forward: GCAATAGATAAAAGAGAAGCGGGAATTATGGTAAAACAACC<br>Reverse: GGTTGTTTTACCATAATTCCCGCTTCTCTTTTATCTATTGC
R443A	Forward: CCATCTACAAGCCAGACAAAGCAGGTGAAGGGTGG<br>Reverse: CCACCCTTCACCTGCTTTGTCTGGCTTGTAGATGG
K449A	Forward: GGTGAAGGGTGGTTAGCAATTAAACCAGAGTATGTCAGTGG<br>Reverse: CCACTGACATACTCTGGTTTAATTGCTAACCACCCTTCACC
K451A	Forward: GGTGAAGGGTGGTTAAAAATTGCACCAGAGTATGTCAGTGGACTAATGG<br>Reverse: CCATTAGTCCACTGACATACTCTGGTGCAATTTTTAACCACCCTTCACC
E546A	Forward: GCATTTTATGTGGAACAGAGAAGCCAGCAGTATACATTGAACCTTGTAATTCTGTC<br>Reverse: GACAGAATTACAAGGTTCAATGTATACTGCTGGCTTCTCTGTTCCACATAAAATGC
R577A	Forward: CTGGCTGCACCTTGGCTTTTCCACGAATTG<br>Reverse: CAATTCGTGGAAAAGCCAAGGTGCAGCCAG
F578A	Forward: CTGGCTGCACCTTGCGTGCTCCACGAATTGAAAAGATAAGAGATGACAAGG<br>Reverse: CCTTGTCATCTCTTATCTTTTCAATTCGTGGAGCACGCAAGGTGCAGCCAG

[1]All primer sequences are oriented 5′ → 3′.

SUPPLEMENTARY METHODS

Mass spectrometry analysis of DNA ligase IV adenylation. 1 μg of purified Ligase IV was digested with 0.05 μg trypsin (Promega) in 25 mM ammonium bicarbonate pH 6.5 for 14 hrs at 25 °C. The digest was desalted by ZipTip (Millipore), essentially using the manufacturer's recommended protocol. ZipTip eluants were spotted (0.3 μL) onto a 192-sample stainless steel MALDI plate and mixed on target with 0.3 μL of 33% saturated α-cyano-hydroxycinnamic acid. Mass spectrometric analyses, MS and MS/MS, were then performed on an AB Sciex 4800 plus MALDI TOF/TOF Analyzer in the positive ion and reflector modes. The MS and MS/MS modes were calibrated externally using either Calmix II (AB Sciex) or the fragment ions of the angiotensin I M+H ion (m/z 1296.68). A focus mass of m/z 2000 was used for the MS acquisition. For the MS/MS, 1 kV was used for the collision energy. Areas under the peak were determined using the Data Explorer software from Applied Biosystems.

Assay for divalent metal dependence of LigIV ligation activity. DNA substrates for ligation assays were prepared by hybridizing an upstream primer (5′-GTCACCTGATGCGTC-3′) and a 5′-phosphorylated, 3'-Cyanine3-labeled downstream primer (5′-pGTCGGACTACTGAGT-Cy3-3′ for unadenylated substrate L4 and 5′-AMP-pGTCGGACTACTGAGT-Cy3-3′ for pre-adenylated substrate L4A) to a dideoxycytidine-terminated template primer (5'-ACTCAGTAGTCCGACGACGCATCAGGTGAC-3') to create a nicked DNA duplex. Reaction mixtures (20 µl) contained 50 mM Tris, pH 8, 1 mM DTT, 4 % glycerol, 0.1 mg/ml BSA, 50 nM DNA substrate, with and without 5 mM $MgCl_2$. 1 mM ATP was added to aid in enzyme adenylation on the unadenylated L4 DNA substrate, and 2 mM EDTA was added to chelate divalent metals in solution, where indicated. Reactions were initiated by adding the catalytic domain construct (residues Met1-Asp620) of wildtype Lig IV at 500 nM and incubating at 37 °C for 1 hour. The reactions were quenched by addition of an equal volume of loading dye (99% (v/v) formamide, 5 mM EDTA, 0.1% (w/v) xylene cyanol, and 0.1% (w/v) bromophenol blue). The products were resolved on a 16% denaturing polyacrylamide gel, imaged using a Typhoon 9400 imager (GE Healthcare Life Sciences).

Screening the effects of divalent metal cations on DNA ligation activity. DNA substrates for ligation assays were prepared by hybridizing an upstream primer (5′-CAGAGTCATAATATGCAGGT-3′) and a 5′-phosphorylated, 3'-TAMRA-labeled downstream primer (5′-pGCAGGTTATTCATGCTCGG-TAMRA-3′) to a template primer (5'-CCGAGCATGAATAACCTGCACCTGCATATTATGACTCTG-3') to create a

nicked DNA duplex. Reaction mixtures (20 µl) contained 50 mM Tris, pH 8, 1 mM DTT, 4 % glycerol, 0.1 mg/ml BSA, 50 nM DNA substrate, 5 mM divalent metal ions. 1 mM ATP was added to aid in enzyme adenylation, where indicated. Reactions were initiated by adding the catalytic domain construct (residues Met1-Asp620) of wildtype Lig IV at 500 nM and incubating at 37 °C for 3 min. The reactions were quenched by addition of an equal volume of loading dye (99% (v/v) formamide, 5 mM EDTA, 0.1% (w/v) xylene cyanol, and 0.1% (w/v) bromophenol blue). The products were resolved on a 16% denaturing polyacrylamide gel, imaged using a Typhoon 9400 imager (GE Healthcare Life Sciences).

SUPPLEMENTARY REFERENCES

1. Ochi, T., Gu, X. & Blundell, T.L. Structure of the catalytic region of DNA ligase IV in complex with an Artemis fragment sheds light on double-strand break repair. *Structure* **21**, 672-9 (2013).
2. Brunger, A.T. et al. Crystallography & NMR system: A new software suite for macromolecular structure determination. *Acta Crystallogr D Biol Crystallogr* **54**, 905-921 (1998).
